# A-site cation manipulation of exemplary second harmonic generation response and optical anisotropy in rare-earth borates†

**DOI:** 10.1039/d4sc05081a

**Published:** 2024-09-06

**Authors:** Jie Song, Huijian Zhao, Conggang Li, Ning Ye, Zhanggui Hu, Yicheng Wu

**Affiliations:** a Tianjin Key Laboratory of Functional Crystal Materials, Institute of Functional Crystal, Tianjin University of Technology Tianjin 300384 China cgli@email.tjut.edu.cn hu@mail.ipc.ac.cn; b State Key Laboratory of Crystal Materials, Shandong University Jinan 250100 China

## Abstract

Ultraviolet nonlinear optical (UV NLO) materials have garnered significant interest for their prospective applications in advanced laser technologies. However, tailoring the desired structure in these materials remains a formidable challenge. Here, we propose a simple yet effective strategy for synthesizing rare-earth borates, K_*x*_Na_3−*x*_La_2_B_3_O_9_ (*x* = 2–3), by manipulating the A-site cations to induce structural evolution. Notably, K_*x*_Na_3−*x*_La_2_B_3_O_9_ undergoes a phase transition from the *Pnc*2 to the *Amm*2 space group by adjusting the K^+^ content to reach *x* = 2.6. Moreover, the target compounds exhibit strong phase-matching second harmonic generation (SHG) efficiencies, ranging from 1.3 to 3.3 times that of KDP (KH_2_PO_4_), and feature short UV cutoff edges of around 204–208 nm. Additionally, the correlation between microscopic polarizability, optical anisotropy, and the structural evolution of these materials was characterized through structural and theoretical analyses. These findings highlight the potential applications of K_*x*_Na_3−*x*_La_2_B_3_O_9_ as UV NLO materials and underscore the viability of manipulating A-site cations to fabricate NLO crystals with desirable properties.

## Abstract 2

Rare-earth borate compounds, K_*x*_Na_3−*x*_La_2_B_3_O_9_ (*x* = 2–3), were designed by finely tuning the composition of A-site cations.

## Graphical abstract

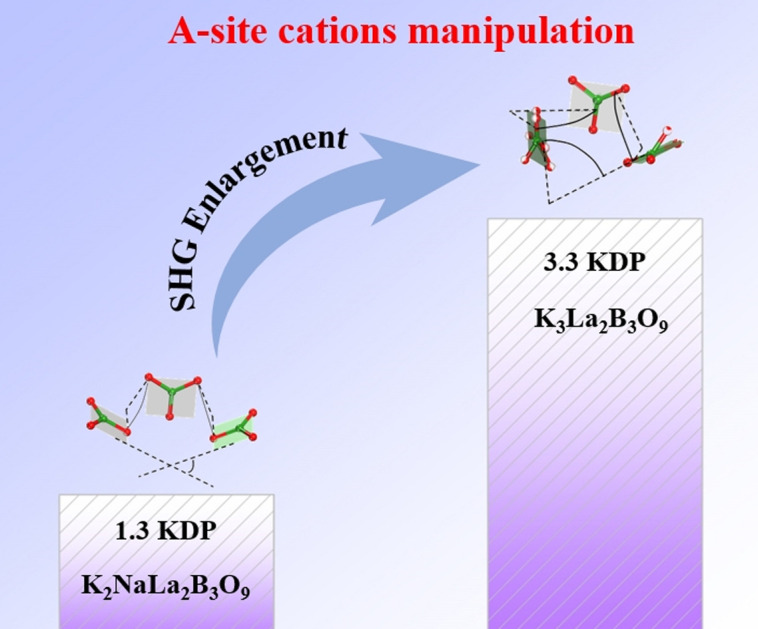

## Introduction

Nonlinear optical (NLO) crystals have garnered considerable attention in scientific and industrial applications due to their ability to manipulate laser frequency and produce coherent light. In particular, ultraviolet (UV) NLO materials have emerged as crucial contenders for extending the wavelength range of solid-state lasers into the UV region, facilitated by a second harmonic generation (SHG) process.^1–3^ These materials hold significant promise in various optoelectronic fields such as laser processing, short-wave communication, semiconductor lithography, biomedicine, and high-density storage.^4–7^ Despite considerable efforts and advancements in exploring acentric materials, the practical realization of UV NLO crystals remains limited. Noteworthy examples include KH_2_PO_4_ (KDP), β-BaB_2_O_4_ (β-BBO), LiB_3_O_5_ (LBO), KBe_2_BO_3_F_2_ (KBBF), and CsLiB_6_O_10_ (CLBO), among others.^8–12^ In response to the development of laser technology, there is an increasing demand for novel NLO crystals capable of directly generating UV coherent light. However, the fabrication of NLO crystals faces challenges stemming from the inherent propensity of inorganic materials to exhibit undesired dipole–dipole interactions or spatial effects, tending to crystallize centrosymmetric structures.^13,14^ Consequently, the development of innovative synthetic strategies to exploit NLO crystals with SHG-enhanced activity remains a significant challenge.

Borates, characterized by their versatile structural configurations and favorable UV transparency attributed to strong covalent B–O bonds, have emerged as a candidate system for exploring UV optical crystals.^15–19^ To effectively innovate and construct novel NLO crystals, the introduction of highly polarizable chromophores into borates is an optional strategy to modify the structure and enhance the optical properties. Strategies such as incorporating second-order Jahn–Teller (SOJT) active cations (Zr^4+^, Nb^5+^, Ta^4+^, *etc.*), stereo-chemically active lone-pair cations (Te^4+^, Bi^3+^, *etc.*), or d^10^ transition metal cations (Zn^2+^, Cd^2+^, *etc.*) have been widely recognized in the quest for new optical materials.^20–22^ Following these design strategies, numerous borates with acentric structures have been synthesized, including BaZr(BO_3_)_2_, CsNbB_2_O_6_, K_3_M_3_B_2_O_12_ (M = Nb, Ta), α-BiB_3_O_6_, CaBi_2_B_2_O_7_, Cd_4_BiO(BO_3_)_3_, Bi_3_TeBO_9_, Na_2_ZnB_6_O_11_, Cs_3_Zn_6_B_9_O_21_, and CaZn_2_(BO_3_)_2_.^2,23–31^ However, the strategies for excavating UV NLO crystals are still limited due to challenges in accurately regulating the structure.

Recent studies have elucidated the indispensable role of alkali metal cations (A-site cations) in the exploration of novel NLO materials, due to the fact that alkali metal cations are devoid of d–d or f–f electron transitions, enabling the broadening of the bandgap and consequently achieving short absorption edges.^32^ Additionally, alkali metal cations with various ionic radii also have a significant impact on the spatial molecular arrangements and macroscopic symmetry. Notable examples include RbB_4_O_6_F (0.8 × KDP), CsB_4_O_6_F (1.9 × KDP), CsRbB_8_O_12_F_2_ (1.1 × KDP), K_7_SrY_2_B_15_O_30_ (1.1 × KDP), Rb_7_SrY_2_(B_5_O_10_)_3_ (0.9 × KDP), Na_3_La_2_(BO_3_)_3_ (2 × KDP), and KNa_2_La_2_(BO_3_)_3_ (2.6 × KDP),^33–38^ exhibiting different NLO activities attributed to the presence of diverse A-site cations. A similar situation has also been observed in alkali metal borophosphate and phosphate UV NLO crystals, such as Rb_3_B_11_P_2_O_23_ (2.5× KDP), Cs_3_B_11_P_2_O_23_ (3 × KDP), α-KZnPO_4_ (0.2 × KDP) and α-LiZnPO_4_ (2.3 × KDP).^39–41^ These investigations suggest that the optimization of local structures induced by A-site cations may enhance SHG performance. However, limited research has been conducted thus far on the evolution process of the structure and performance of distinct A-site cations in these NLO borate-based derivatives.

To tackle this challenge, we utilized rare earth borates as a foundation and introduced alkali metals with varying ionic radii to optimize the structural arrangement and optical properties by adjusting the composition of alkali metals. For instance, the substitution of Na^+^ and K^+^ cations is widely regarded as a viable approach due to the inherent flexibility in doping between these components.^42^ Additionally, the incorporation of rare earth cations, like Sc^3+^, Y^3+^, La^3+^, Gd^3+^ and Lu^3+^, not only contributes to excellent transparency in the UV region, but also offers various coordination types.^43^ Notably, the La^3+^ cation with a sizable ionic radius tends to form flexible SHG-active chromophores, thereby generating favorable SHG effects.^38^ Inspired by these ideas, our work focuses on rare earth borate-based materials and explores the underlying mechanism of the NLO effect and optical anisotropy by employing a facile strategy to manipulate the components of A-site alkali metal cations. The synthesis of rare-earth borates K_*x*_Na_3−*x*_La_2_B_3_O_9_ (*x* = 2–3) was achieved through structural evolutions attributed to A-site cation manipulations. As anticipated, these compounds displayed remarkable phase-matching SHG efficiencies ranging from 1.3 to 3.3 times that of KDP, while featuring short UV cutoff edges of approximately 204 nm. These findings highlight the potential of K_*x*_Na_3−*x*_La_2_B_3_O_9_ as favorable candidates for UV NLO applications and underscore the significance of regulating A-site alkali metal cations in the exploration of new NLO crystals.

## Results and discussion

### Synthesis and phase transformation

**Fig. 1:**
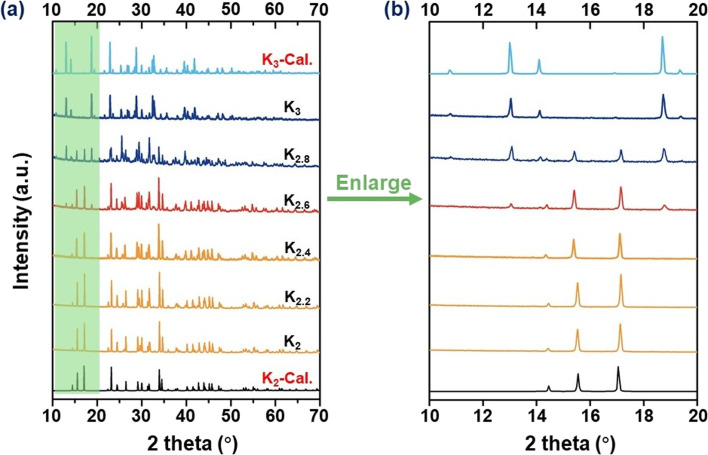
(a) Experimental and calculated PXRD patterns for K_2_, K_2.2_, K_2.4_, K_2.6_, K_2.8_, and K_3_ polycrystals, respectively. (b) The enlarged PXRD curves observed in the 2*θ* range of 10–20°.

The preparation of the target compounds was performed through a modified solid-phase reaction. To monitor the influence of A-site cations on the crystal structure, a series of compounds with varying proportions of A-site cations, namely K_2_, K_2.2_, K_2.4_, K_2.6_, K_2.8_, and K_3_, were synthesized. The phase purity of the resulting products was verified using PXRD analysis. It is evident from Fig. 1a that the K_2_ and K_3_ compounds demonstrate distinct crystal structural characteristics. Specifically, the structural characteristics of K_2.8_ and K_3_ exhibited isomorphism, suggesting a consistent structural framework despite the varying proportions of K cations in the A-site. However, when the K^+^ cation proportion reached approximately K_2.6_, structural features indicative of the K_2_ compound were initially observed, implying the coexistence of two distinct polycrystalline phases. This observation confirms that the structural transition from K_3_ to K_2_ occurs at around the K_2.6_ proportion. Furthermore, the PXRD peak patterns of K_2_, K_2.2_, and K_2.4_ displayed a high degree of similarity, suggesting isomorphism between these compounds. These observations provide clear evidence of the critical impact of the A-site cation proportions on the structural regulation of these materials. Notably, as illustrated in Fig. 1b, an increase in the K proportion within the range of K_2_–K_2.4_ leads to a gradual shift of PXRD peaks towards lower angles, indicating larger lattice constants.^44^ This trend is also observed when considering the proportion of K variation within the K_2.8_–K_3_ range. These findings strongly suggest that the proportion of K^+^ cations exerts a direct impact on the lattice constants of these compounds.

### Thermal analyses

**Fig. 2:**
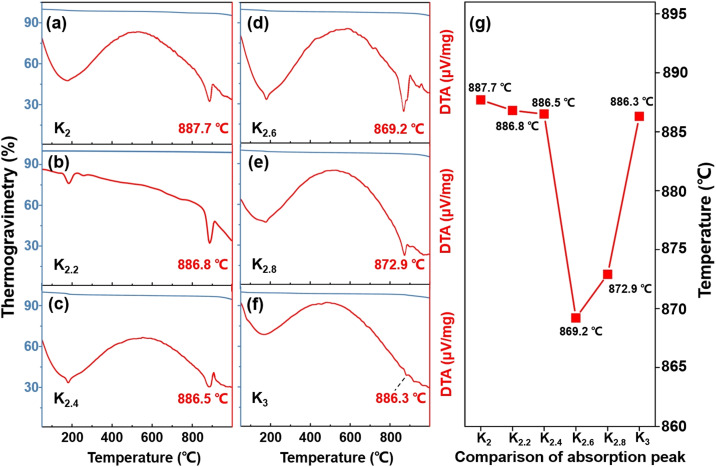
(a–f) TG-DTA curves for the K_2_, K_2.2_, K_2.4_, K_2.6_, K_2.8_, and K_3_ compounds, respectively. (g) Comparative results of thermal stabilities for the title compounds.

Thermal analyses were conducted to further investigate the structural transformations and evaluate the thermal stability of the title compounds. TG-DTA measurements revealed the remarkable thermal stability of the K_2_, K_2.2_, K_2.4_, K_2.6_, K_2.8_, and K_3_ compounds, as evidenced by sharp absorption peaks observed above 850 °C (Fig. 2a). Notably, the thermal stability of compounds within the K_2_–K_2.6_ range exhibited a decreasing trend with increasing proportions of K^+^ cations (Fig. 2b). In contrast, within the K_2.6_–K_3_ range, the compounds displayed an upward trend in thermal stability (Fig. 2g). Among the tested compound systems, it has been observed that the K_2.6_ compound demonstrated the lowest thermal stability. When the K^+^ cation reaches a composition of 2.6, this behavior can potentially be attributed to the influence of mixed components present in the system that cannot be ignored, leading to a decrease in the melting point of the system. These findings further highlight the role of the K_2.6_ compound, which possesses mixed-phase structures that align with the PXRD analyses.

### Crystal structures of K_2_ and K_3_

**Fig. 3:**
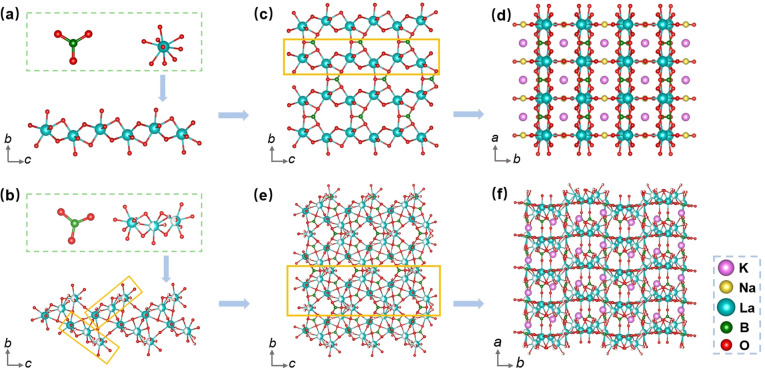
Structural characteristics of K_2_ and K_3_. (a and b) [BO_3_] plane triangles, [LaO_9_] polyhedra, and [La_2_O_16_]_∞_ and [La_3_O_21_] clusters. (c) Pseudo-2D layers of K_2_. (d) 3D structural framework of K_2_. (e) Presentation of [La_3_O_21_]_∞_ clusters connected with [BO_3_] units in K_3_ viewed from the *bc* plane. (f) 3D structural network of K_3_ viewed along the *c*-axis.

In light of the isomorphism observed among the K_2_, K_2.2_, and K_2.4_ compounds, as well as the isomorphism between the K_2.8_ and K_3_ compounds, we have selected K_2_ and K_3_ as representative samples for conducting a thorough analysis of their respective crystal structures. The structural features of K_2_ and K_3_ were determined through the utilization of single-crystal XRD analysis (Tables S1–S5†). Our analysis revealed that K_2_ adopts an asymmetric orthorhombic crystal structure, belonging to the space group *Amm*2 (no. 38), while K_3_ exhibits a similar orthorhombic structure in the *Pnc*2 space group (no. 30), as summarized in Table S1.† In the crystal structure of K_2_, the asymmetric unit consists of two distinct K, one Na, one La, two B, and four O atoms. Conversely, the asymmetric unit of K_3_ contains three K, three La, four B, and eleven O atoms. As shown in Fig. 3a and b, the B atoms are coordinated by three O atoms, forming [BO_3_] plane triangles, and the La atom is surrounded by nine O atoms, resulting in the formation of distorted [LaO_9_] polyhedra. The B–O bond lengths in K_2_ range from 1.360(2) to 1.379(12) Å, while the La–O bond lengths range from 2.466(12) to 2.687(5) Å. In K_3_, the B–O bond lengths vary from 1.300(5) to 1.409(15) Å, whereas the La–O bond lengths range from 2.412(8) to 3.02(2) Å. In K_2_, adjacent [LaO_9_] polyhedra are connected to each other through oxygen edge-sharing, forming a pseudo-one-dimensional (1D) chain denoted as [La_2_O_16_]_∞_. These 1D chains, along with [BO_3_] units, further interconnect through oxygen corner-sharing to form pseudo-two-dimensional (2D) layers on the *bc* plane (Fig. 3c). These pseudo-2D layers are then bridged with [BO_3_] units along the *a*-axis through oxygen corner-sharing, ultimately giving rise to a three-dimensional (3D) structural framework (Fig. 3d). In the case of K_3_, three distorted [LaO_9_] polyhedra associate to form distinct [La_3_O_21_] clusters *via* oxygen face-sharing. These clusters are further interconnected with [BO_3_] units by sharing oxygen along the *bc* plane, as presented in Fig. 3e, with the K^+^ cations located in the channels (Fig. 3f).

**Fig. 4:**
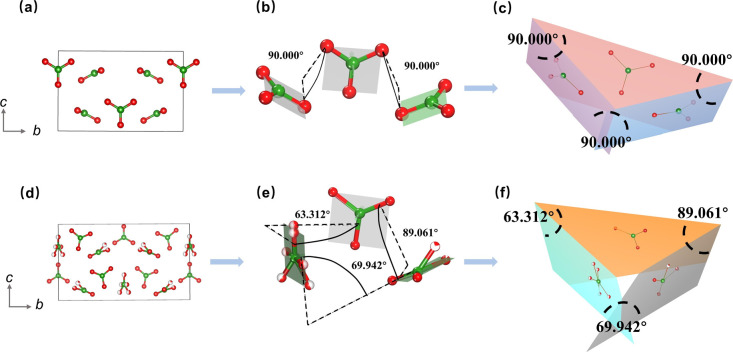
Comparison of the arrangements of distinct [BO_3_] units in the K_2_ and K_3_ compounds. (a–c) Arrangements and orientations of the isolated [BO_3_] units in the K_2_ compound. (d–f) Arrangements and orientations of the isolated [BO_3_] units in the K_3_ compound.

To shed light on the structural evolution exhibited by the K_2_ and K_3_ compounds, our study focuses on a thorough analysis of the arrangement and interrelationships of fundamental [BO_3_] units. Notably, while both K_2_ and K_3_ compounds feature isolated planar triangles formed by [BO_3_] units, a notable disparity arises in the spatial arrangement of these triangles within their respective 3D frameworks. As shown in Fig. 4a–c, it is noteworthy that the two distinct [BO_3_] plane triangles in K_2_ exhibit a regular arrangement, manifesting a constant angle of 90 degrees between the planes they occupy. In contrast, the planes defined by the distinct [BO_3_] units in K_3_ feature varying angles, including 63.312, 89.061, and 69.942 degrees, which deviate significantly from the uniform 90 degree arrangement observed in K_2_. The variations in the spatial angles between the [BO_3_] building blocks in K_2_ and K_3_ have distinct implications for the anisotropic characteristics of their respective structures. These observations would contribute to a more comprehensive understanding of the potential structural and application-related implications associated with the different spatial arrangements of [BO_3_] units in K_2_ and K_3_.

### Spectroscopic properties

**Fig. 5:**
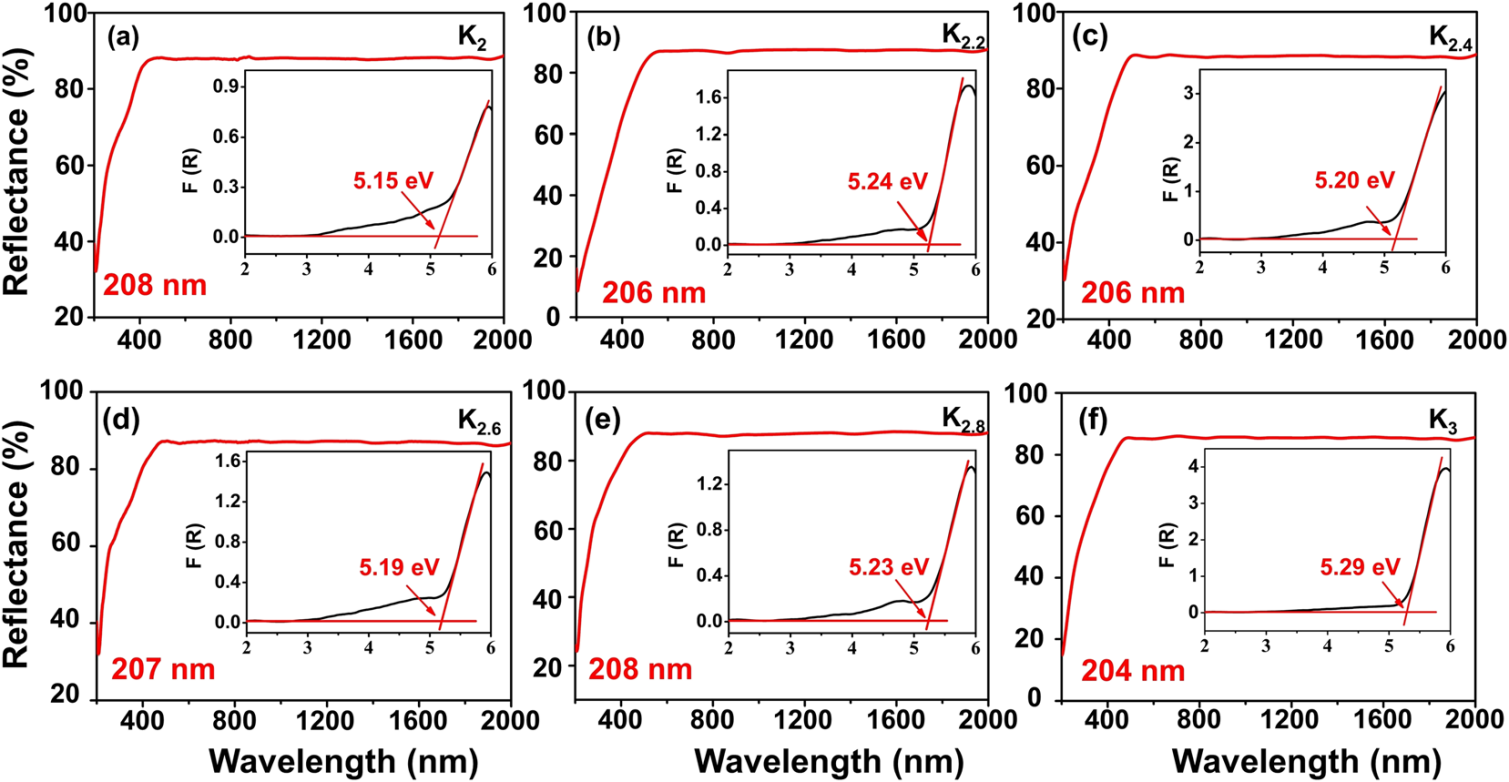
(a–f) UV-vis-NIR diffuse reflectance spectra for the K_2_, K_2.2_, K_2.4_, K_2.6_, K_2.8_, and K_3_ polycrystalline samples, respectively.

To investigate the optical properties of the title compounds, the UV-vis-NIR diffuse reflectance spectra of the title compounds were recorded under consistent conditions (Fig. 5). The obtained spectra revealed distinct UV transmittance cut-off edges for each compound: 208, 206, 206, 207, 208, and 204 nm for the K_2_, K_2.2_, K_2.4_, K_2.6_, K_2.8_, and K_3_ compounds, respectively. These favorable UV transmittance characteristics directly correspond to energy band gaps of 5.15, 5.24, 5.20, 5.19, 5.23, and 5.29 eV, respectively, as determined using the Kubelka–Munk formula.^45^ Furthermore, we conducted a detailed analysis of the infrared (IR) spectra of the compounds, which revealed similar characteristics among them, as depicted in Fig. S1.† Taking the K_2_ compound as an example, the absorption peaks observed at around 1392.73 and 1200.69 cm^−1^ are attributed to the asymmetric stretching vibrations of the [BO_3_] units, while the peak near 950.08 cm^−1^ corresponds to the symmetric stretching vibrations. The peaks around 743.24 and 604.83 cm^−1^ are caused by the bending vibrations of the [BO_3_] units. The presence of these distinct vibration modes further confirms the existence of the [BO_3_] unit within the compounds.^37,38,43*b*^ Notably, we observed a slight redshift phenomenon in the absorption peak positions of the IR spectrum with an increasing K to Na component ratio. This trend can primarily be attributed to the expansion of the alkali metal cation radius.^46^ With a larger cation radius, the bond length between the cation and oxygen atom increases, resulting in a lower vibration frequency and a consequent redshift in the absorption peak position of the IR absorption spectrum. These findings provide valuable insights into the intrinsic relationship between structural changes and the optical properties exhibited by the investigated compounds.

### SHG characterization

**Fig. 6:**
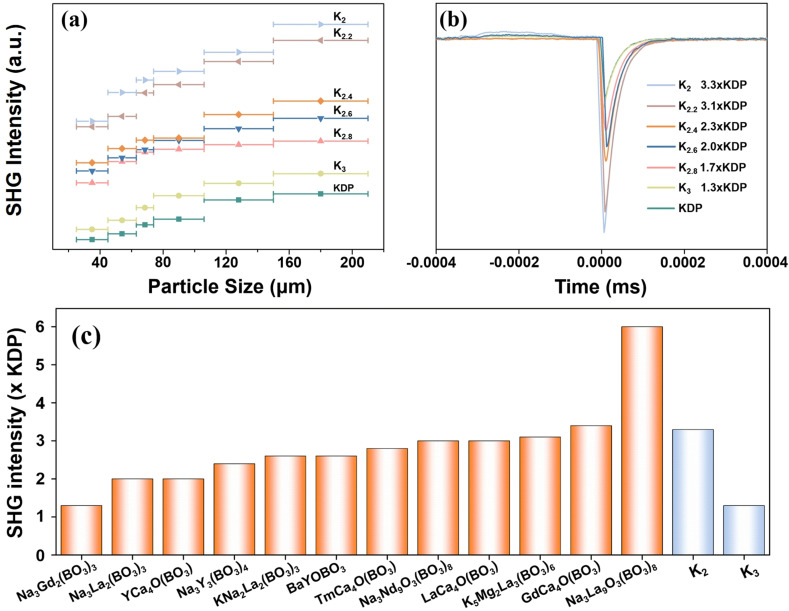
(a) Phase-matched curves for KDP, K_2_, K_2.2_, K_2.4_, K_2.6_, K_2.8_, and K_3_ polycrystalline powders under 1064 nm laser irradiation. (b) Comparison of SHG intensity between the KDP reference and the title compounds. (c) Comparison of the reported SHG intensity of alkali/alkaline earth metal rare earth borates containing [BO_3_] units.

The title compounds are considered to possess SHG responses because they crystallize in non-centrosymmetric space groups. Fig. 6a presents a plot illustrating the correlation between the particle size and the detected powder SHG signals. It is observed that the SHG response intensity of each compound increases with increasing particle size until it reaches its maximum value, suggesting phase-matching behavior in accordance with the rules proposed by Kurtz and Perry.^47^

Furthermore, the SHG intensities of K_2_, K_2.2_, K_2.4_, K_2.6_, K_2.8_, and K_3_ were determined to be 3.3, 3.1, 2.3, 2.0, 1.7, and 1.3 times that of the benchmark KDP, respectively, within a particle size range of 177 to 210 μm (Fig. 6b). The SHG responses are sufficiently large for UV NLO applications. Notably, the significant disparity in the SHG effect between K_2_ and K_3_ can primarily be attributed to the disordered arrangement of oxygen atoms in K_3_, which gives rise to an inconsistent alignment of the [BO_3_] triangular units, consequently resulting in a lower microscopic second-order hyper-polarizability compared to K_2_.^48^ Additionally, as the ratio of K to Na in these compounds gradually increases, the SHG response correspondingly decreases. This observation may be closely related to the unit cell volume of the crystal structure, where a larger unit cell volume tends to weaken the second-order polarizability. Fig. 6c and Table S6† provide a compilation of the reported SHG intensities of alkali and alkaline earth metal borate compounds containing [BO_3_] triangular units from the literature.^49^ Notably, K_2_ exhibits a significant SHG response compared with other reported compounds, thereby bolstering its potential as a NLO crystal.

### Birefringence characterization

**Fig. 7:**
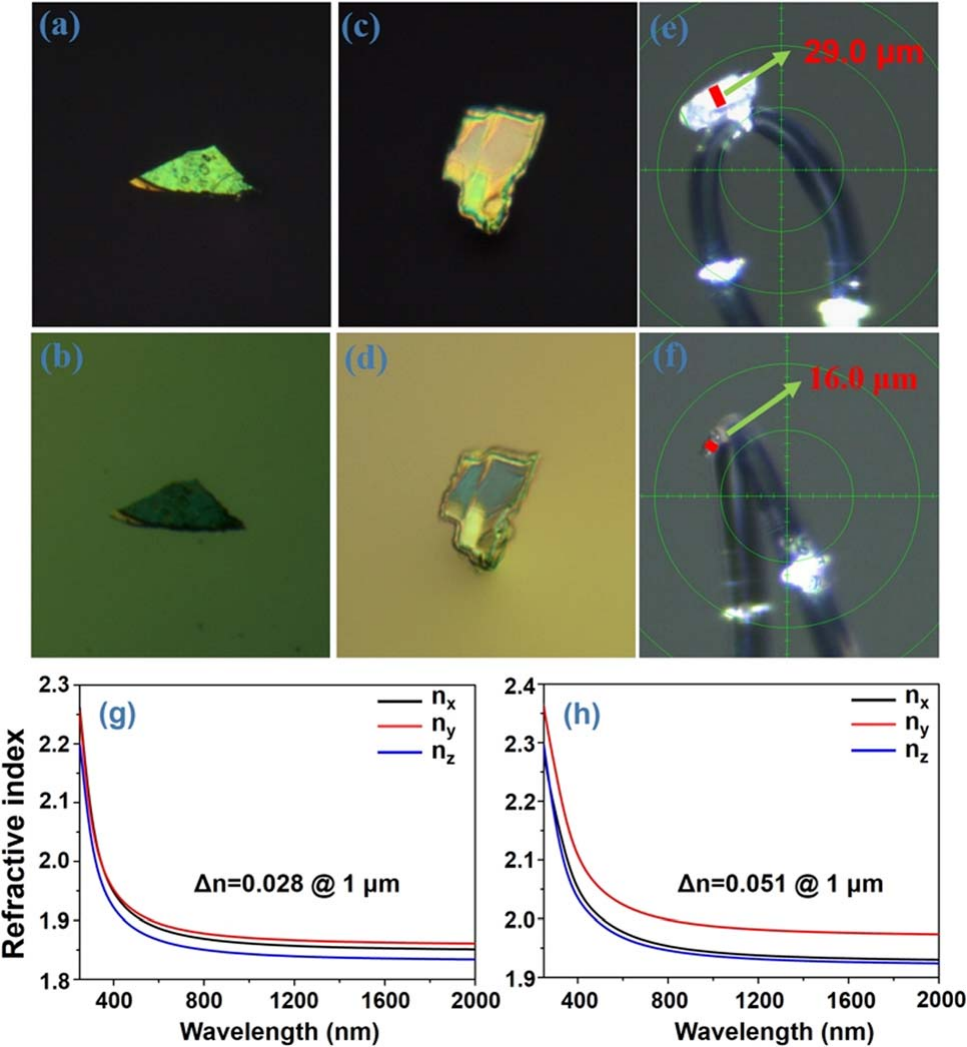
(a–d) Original interference state and complete extinction of K_2_ and K_3_ crystals under cross-polarized light, respectively. (e and f) The thickness of the K_2_ and K_3_ crystals, respectively. (g and h) Theoretical dispersion curve of the refractive index for K_2_ and K_3_ crystals, respectively.

The birefringence of K_2_ and K_3_ crystals was evaluated within the visible wavelength range using a polarizing microscope. Fig. 7a–d show the complete extinction of both K_2_ and K_3_ crystals under orthogonally polarized light. The measured crystal thicknesses (*d*) were found to be 29 μm and 16 μm, corresponding to the optical path differences (*R*) of 841 nm and 896 nm,^50^ respectively. Consequently, the birefringence values of K_2_ and K_3_ crystals were determined to be 0.029 and 0.056, respectively, across the visible wavelength range. The theoretical birefringent values of the two crystals were also calculated, yielding values of 0.028 and 0.051 for K_2_ and K_3_ crystals (Fig. 7g and h), respectively, at a wavelength of 1 μm, which aligns with the experimental observations. Notably, the birefringence value of K_3_ is significantly higher than that of K_2_, nearly double that of K_2_. This significant difference primarily stems from the angles between the [BO_3_] planes in K_2_ and K_3_, as discussed in the “Crystal structure” section. Specifically, the spatial structure of K_2_ consists of two distinct perpendicular [BO_3_] units oriented at a 90 degree angle to each other, indicating relatively small structural anisotropy. In contrast, K_3_ displays an arrangement in which the [BO_3_] units form varying angles, including 63.312, 89.061, and 69.942 degrees, thereby deviating from perpendicularity. This distinct structural feature in K_3_ results in relatively large structural anisotropy.^29^

### Structure–property correlations

**Fig. 8:**
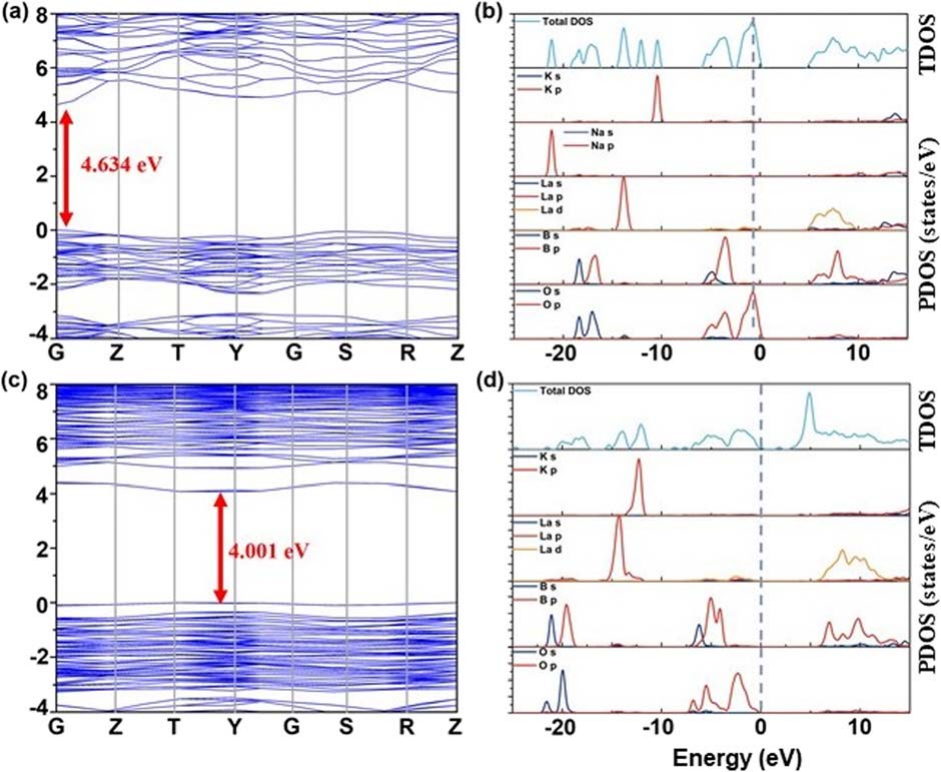
(a and b) The presentation of the band structure as well as the PDOS and TDOS for K_2_, respectively. (c and d) The presentation of the band structure as well as the PDOS and TDOS for K_3_, respectively.

To delve deeper into the electronic structures of K_2_ and K_3_ crystals and their inherent relationships with optical properties, this study employed DFT-based computational methods for theoretical research.^51^ Through first-principles calculations,^52^ we discovered that both K_2_ and K_3_ crystals are direct bandgap compounds, indicating that the lowest point of the conduction band and the highest point of the valence band overlap in momentum space (*k*-space). The calculated theoretical bandgap values for K_2_ and K_3_ crystals are 4.634 eV and 4.001 eV, respectively, as shown in Fig. 8a–c. The orbital contributions of each atom to the energy bands of K_2_ and K_3_ can be identified from the corresponding partial densities of states (PDOSs). As shown in Fig. 8b–d, below the Fermi level, the valence band maximum predominantly consists of B 2p and O 2p orbitals, while the conduction band minimum primarily originates from the La 5d orbitals in both K_2_ and K_3_ compounds. It is noteworthy that the electron states in K_2_ predominantly occupied by K 3p orbitals range from −12 to −11 eV, whereas those in K_3_, governed by K 3p orbitals, span from −13 to −11 eV. Given the close correlation between the optical properties and the optical transitions occurring between electronic states in proximity to the band gap, it can be deduced that the optical activities of the K_2_ and K_3_ crystals are primarily influenced by the [BO_3_] units and [LaO_9_] polyhedra.

## Conclusions

In summary, a new family of alkaline-metal rare-earth borates, K_*x*_Na_3−*x*_La_2_B_3_O_9_ (*x* = 2–3), was synthesized through A-site cation control engineering. The investigation of these compounds *via* DTA-TG analyses and PXRD tests revealed a structural transition from the *Pnc*2 to the *Amm*2 space group. Notably, the structural phase of the K_*x*_Na_3−*x*_La_2_B_3_O_9_ compounds was found to be modulated by adjusting the A-site cation K^+^ content. Intriguingly, these materials display large SHG intensities, which are 1.3 to 3.3 times that of KDP with phase-matchable ability, along with short UV absorption edges at approximately 204–208 nm. Moreover, the K_2_NaLa_2_B_3_O_9_ and K_3_La_2_B_3_O_9_ crystals selected for analysis possess moderate birefringence values of 0.029 and 0.056, respectively, with the variation primarily attributed to differing orientations of the [BO_3_] planes. These favorable findings make K_2_NaLa_2_B_3_O_9_ and K_3_La_2_B_3_O_9_ good potential candidates for UV NLO applications. The rational regulation of A-site cations underscores a versatile approach for the fabrication of novel NLO crystals with desired performance attributes.

## Author contributions

Jie Song and Huijian Zhao: experiment, investigation, data curation, writing original draft. Conggang Li: conceptualization, funding acquisition, methodology, project administration, review & editing. Ning Ye, Zhanggui Hu and Yicheng Wu: resources, funding acquisition.

## Conflicts of interest

There are no conflicts to declare.

## Supplementary Material

SC-015-D4SC05081A-s001

SC-015-D4SC05081A-s002

## Data Availability

The data supporting this article have been included as part of the ESI.† Crystallographic data for 2374500 and 2374501 have been deposited at the CCDC.

## References

[cit1] Eaton D. F. (1991). Nonlinear Optical Materials. Science.

[cit2] Xia M. J., Jiang X. X., Lin Z. S., Lit R. K. (2016). “All-Three-in-One”: A New Bismuth-Tellurium-Borate Bi_3_TeBO_9_ Exhibiting Strong Second Harmonic Generation Response. J. Am. Chem. Soc..

[cit3] Chen X. L., Zhang B. B., Zhang F. F., Wang Y., Zhang M., Yang Z. H., Poeppelmeier K. R., Pan S. L. (2018). Designing an Excellent Deep-Ultraviolet Birefringent Material for Light Polarization. J. Am. Chem. Soc..

[cit4] Halasyamani P. S., Rondinelli J. M. (2018). The must-have and nice-to-have experimental and computational requirements for functional frequency doubling deep-UV crystals. Nat. Commun..

[cit5] Wu C., Jiang C. B., Wei G. F., Jiang X. X., Wang Z. J., Lin Z. S., Huang Z. P., Humphrey M. G., Zhang C. (2023). Toward Large Second-Harmonic Generation and Deep-UV Transparency in Strongly Electropositive Transition Metal Sulfates. J. Am. Chem. Soc..

[cit6] Zou G. H., Ok K. M. (2020). Novel ultraviolet (UV) nonlinear optical (NLO) materials discovered by chemical substitution-oriented design. Chem. Sci..

[cit7] Pan C. Y., Yang X. R., Xiong L., Lu Z. W., Zhen B. Y., Sui X., Deng X. B., Chen L., Wu L. M. (2020). Solid-State Nonlinear Optical Switch with the Widest Switching Temperature Range Owing to Its Continuously Tunable *T*c. J. Am. Chem. Soc..

[cit8] Zaitseva N., Carman L., Smolsky I. (2002). Habit control during rapid growth of KDP and DKDP crystals. J. Cryst. Growth.

[cit9] Miyazaki K., Sakai H., Sato T. (1986). Efficient deep-ultraviolet generation by frequency doubling in β-BaB_2_O_4_ crystals. Opt. Lett..

[cit10] Wang Q., Yang F., Wang X., Zhou J., Ju J., Huang L., Gao D. J., Bi J., Zou G. H. (2019). Deep-Ultraviolet Mixed-Alkali-Metal Borates with Induced Enlarged Birefringence Derived from the Structure Rearrangement of the LiB_3_O_5_. Inorg. Chem..

[cit11] Wu B., Tang D., Ye N., Chen C. (1996). Linear and nonlinear optical properties of the KBe_2_BO_3_F_2_ (KBBF) crystal. Opt. Mater..

[cit12] Mori Y., Kuroda I., Nakajima S., Sasaki T., Nakai S. (1995). New nonlinear optical crystal: Cesium lithium borate. Appl. Phys. Lett..

[cit13] Kang L., Lin Z. S. (2022). Deep-ultraviolet nonlinear optical crystals: concept development and materials discovery. Light: Sci. Appl..

[cit14] Valdivia-Berroeta G. A., Jackson E. W., Kenney K. C., Wayment A. X., Tangen I. C., Bahr C. B., Smith S. J., Michaelis D. J., Johnson J. A. (2020). Designing Non-Centrosymmetric Molecular Crystals: Optimal Packing May Be Just One Carbon Away. Adv. Funct. Mater..

[cit15] Mutailipu M., Poeppelmeier K. R., Pan S. L. (2021). Borates: A Rich Source for Optical Materials. Chem. Rev..

[cit16] Song J. L., Hu C. L., Xu X., Kong F., Mao J. G. (2015). A Facile Synthetic Route to a New SHG Material with Two Types of Parallel π-Conjugated Planar Triangular Units. Angew. Chem., Int. Ed..

[cit17] Huppertz H., von der Eltz B. (2002). Multianvil high-pressure synthesis of Dy_4_B_6_O_15_: the first oxoborate with edge-sharing BO_4_ tetrahedra. J. Am. Chem. Soc..

[cit18] Sohr G., Ciaghi N., Schauperl M., Wurst K., Liedl K. R., Huppertz H. (2015). High-Pressure Synthesis of Cd(NH_3_)_2_[B_3_O_5_(NH_3_)]_2_: Pioneering the Way to the Substance Class of Ammine Borates. Angew. Chem., Int. Ed..

[cit19] Kong F., Huang S. P., Sun Z. M., Mao J. G., Cheng W. D. (2006). Se_2_(B_2_O_7_): a new type of second-order NLO material. J. Am. Chem. Soc..

[cit20] Lu W. Q., Gao Z. L., Liu X. T., Tian X. X., Wu Q., Li C. G., Sun Y. X., Liu Y., Tao X. T. (2018). Rational Design of a LiNbO_3_-like Nonlinear Optical Crystal, Li_2_ZrTeO_6_, with High Laser-Damage Threshold and Wide Mid-IR Transparency Window. J. Am. Chem. Soc..

[cit21] Chen H., Wei W. B., Lin H., Wu X. T. (2021). Transition-metal-based chalcogenides: a rich source of infrared nonlinear optical materials. Coord. Chem. Rev..

[cit22] Chen Y. N., Zhang M., Mutailipu M., Poeppelmeier K. R., Pan S. L. (2019). Research and Development of Zincoborates: Crystal Growth, Structural Chemistry and Physicochemical Properties. Mol.

[cit23] Schultze D., Wilke K.-T., Waligora C. (1971). Zur Chemie in Schmelzlösungen. IV. Darstellung von kristallinen Boraten M^2+^M^4+^(BO_3_)_2_. Z. Anorg. Allg. Chem..

[cit24] Akella A., Keszler D. A. (1995). Crystal Chemistry of Noncentrosymmetric Alkali-Metal Nb and Ta Oxide Pyroborates. J. Solid State Chem..

[cit25] Choisnet J., Groult D., Raveau B., Gasperin M. (1977). Nouvelles structures à tunnels de section pentagonale K_3_Nb_3_B_2_O_12_ et K_3_Ta_3_B_2_O_12_. Acta Crystallogr..

[cit26] Zhang K., Chen X., Wang X. (2005). Review of Study on Bismuth Triborate (BiB_3_O_6_) Crystal. J. Synth. Cryst..

[cit27] Barbier J., Cranswick L. M. D. (2006). The non-centrosymmetric borate oxides, MBi_2_B_2_O_7_ (M = Ca, Sr). J. Solid State Chem..

[cit28] Zhang W. L., Cheng W. D., Zhang H., Geng L., Lin C. S., He Z. Z. (2010). A Strong Second-Harmonic Generation Material Cd_4_BiO(BO_3_)_3_ Originating from 3-Chromophore Asymmetric Structures. J. Am. Chem. Soc..

[cit29] Mutailipu M., Li F. M., Jin C. C., Yang Z. H., Poeppelmeier K. R., Pan S. L. (2022). Strong Nonlinearity Induced by Coaxial Alignment of Polar Chain and Dense BO_3_ Units in CaZn_2_(BO_3_)_2_. Angew. Chem., Int. Ed..

[cit30] Chen Y. Q., Liang J. K., Gu Y. X., Luo J., Li J. B., Rao G. H. (2010). Synthesis and crystal structure of a novel hexaborate, Na_2_ZnB_6_O_11_. Powder Diffr..

[cit31] Yu H. W., Wu H. P., Pan S. L., Yang Z. H., Hou X. L., Su X., Jing Q., Poeppelmeier K. R., Rondinelli J. M. (2014). Cs_3_Zn_6_B_9_O_21_: A Chemically Benign Member of the KBBF Family Exhibiting the Largest Second Harmonic Generation Response. J. Am. Chem. Soc..

[cit32] Meng X. H., Zhang X. Y., Liu Q. X., Zhou Z. Y., Jiang X. X., Wang Y. G., Lin Z. S., Xia M. J. (2023). Perfectly Encoding π-Conjugated Anions in the RE_5_(C_3_N_3_O_3_)(OH)_12_ (RE = Y, Yb, Lu) Family with Strong Second Harmonic Generation Response and Balanced Birefringence. Angew. Chem., Int. Ed..

[cit33] Yang Z. H., Tudi A., Lei B. H., Pan S. L. (2020). Enhanced nonlinear optical functionality in birefringence and refractive index dispersion of the deep-ultraviolet fluorooxoborates. Sci. China Mater..

[cit34] Wang Y., Zhang B. B., Yang Z. H., Pan S. L. (2018). Cation-Tuned Synthesis of Fluorooxoborates: Towards Optimal Deep-Ultraviolet Nonlinear Optical Materials. Angew. Chem., Int. Ed..

[cit35] Mutailipu M., Xie Z. Q., Su X., Zhang M., Wang Y., Yang Z. H., Janjua M., Pan S. L. (2017). Chemical Cosubstitution-Oriented Design of Rare-Earth Borates as Potential Ultraviolet Nonlinear Optical Materials. J. Am. Chem. Soc..

[cit36] Li Y. F., Liang F., Song H. M., Liu W., Lin Z. S., Zhang G. C., Wu Y. C. (2019). Rb_7_SrY_2_(B_5_O_10_)_3_: A Rare-Earth Pentaborate with Moderate Second-Harmonic Response and Interesting Phase-Matching Behavior. Inorg. Chem..

[cit37] Zhang G. C., Wu Y. C., Fu P. Z., Wang G. F., Pan S. L., Chen C. T. (2001). A new nonlinear optical berate crystal Na_3_La_2_(BO_3_)_3_. Chem. Lett..

[cit38] Song J., Li C. G., Jiao J. M., She Y. H., Zhao W. L., Liang F., Ye N., Hu Z. G., Wu Y. C. (2023). KNa_2_La_2_(BO_3_)_3_: a shortite-type lanthanide borate exhibiting strong nonlinear optical activity induced by isolated BO_3_ triangles and distorted LaO_9_ polyhedra. Inorg. Chem. Front..

[cit39] Liu H. N., Wu H. P., Hu Z. G., Wang J. Y., Wu Y. C., Halasyamani P. S., Yu H. W. (2023). Rb_3_B_11_P_2_O_23_: Materials Design of a New Chemically Benign Deep-Ultraviolet Nonlinear Optical Material. ACS Mater. Lett..

[cit40] Liu H. A., Wu H. P., Hu Z. G., Wang J. Y., Wu Y. C., Yu H. W. (2023). Cs_3_(BOP)_2_(B_3_O_7_)_3_: A Deep-Ultraviolet Nonlinear Optical Crystal Designed by Optimizing Matching of Cation and Anion Groups. J. Am. Chem. Soc..

[cit41] He X. M., Qi L., Zhang W. Y., Zhang R. X., Dong X. Y., Ma J. H., Abudoureheman M., Jing Q., Chen Z. H. (2023). Controlling the Nonlinear Optical Behavior and Structural Transformation with A-Site Cation in α-AZnPO_4_ (A = Li, K). Small.

[cit42] Han S. J., Wang Y., Pan S. L., Dong X. Y., Wu H. P., Han J., Yang Y., Yu H. W., Bai C. Y. (2014). Noncentrosymmetric *versus* Centrosymmetric: Influence of the Na^+^ Substitution on Structural Transition and Second-Harmonic Generation Property. Cryst. Growth Des..

[cit43] Wu Y. C., Liu J. G., Fu P. Z., Wang J. X., Zhou H. Y., Wang G. F., Chen C. T. (2001). A new lanthanum and calcium borate La_2_CaB_10_O_19_. Chem. Mater..

[cit44] H awes L. L. (1959). The determination of lattice constants using low angle diffraction lines. Acta Crystallogr..

[cit45] Kubelka P., Munk F. (1931). An Article on Optics of Paint Layers. Zeitschrift für Technische Physik.

[cit46] He F., Zhuang J. H., Lu B., Liu X. L., Zhang J. L., Gu F. N., Zhu M. H., Xu J., Zhong Z. Y., Xu G. W., Su F. B. (2021). Ni-based catalysts derived from Ni-Zr-Al ternary hydrotalcites show outstanding catalytic properties for low-temperature CO_2_ methanation. Appl. Catal., B.

[cit47] Kurtz S. K., Perry T. T. (1968). A Powder Technique for the Evaluation of Nonlinear Optical Materials. J. Appl. Phys..

[cit48] Li X. Y., Wei Q., Hu C. L., Pan J., Li B. X., Xue Z. Z., Li X. Y., Li J. H., Mao J. G., Wang G. M. (2023). Achieving Large Second Harmonic Generation Effects *via* Optimal Planar Alignment of Triangular Units. Adv. Funct. Mater..

[cit49] Zhang G. C., Wu Y. C., Li Y. G., Chang F., Pan S. L., Fu P. Z., Chen C. T. (2005). Flux growth and characterization of a new oxyborate crystal Na_3_La_9_O_3_(BO_3_)_8_. J. Cryst. Growth.

[cit50] Huang W. Q., Zhang X., Li Y. Q., Zhou Y., Chen X., Li X. Q., Wu F. F., Hong M. C., Luo J. H., Zhao S. G. (2022). A Hybrid Halide Perovskite Birefringent Crystal. Angew. Chem., Int. Ed..

[cit51] Godby R. W., Schlüter M., Sham L. J. (1988). Self-energy operators and exchange-correlation potentials in semiconductors. Phys. Rev. B: Condens. Matter Mater. Phys..

[cit52] Clark S. J., Segall M. D., Pickard C. J., Hasnip P. J., Probert M. J., Refson K., Payne M. C. (2005). First principles methods using CASTEP. Z. Kristallogr..

