# Supplementary material for: A-site cation manipulation of exemplary second harmonic generation response and optical anisotropy in rare-earth borates
Source: Chem Sci. 2024 Sep 6;15(39):16196–204. doi: 10.1039/d4sc05081a (PMC11403946; doi:10.1039/d4sc05081a)
Supplement: SC-015-D4SC05081A-s001 [file SC-015-D4SC05081A-s001.pdf]

Supplementary Information (SI) for Chemical Science.
This journal is © The Royal Society of Chemistry 2024

Supporting Information

A-site cations manipulation of exemplary second harmonic generation response and optical anisotropy in rare-earth borates

Jie Song,[#,a] Huijian Zhao,[#,a] Conggang Li,*[,a,b] Ning Ye,[a] Zhanggui Hu,*[,a] and Yicheng Wu[a]

[a]*Tianjin Key Laboratory of Functional Crystal Materials, Institute of Functional Crystal, Tianjin University of Technology, Tianjin 300384, China.*

[b]*State Key Laboratory of Crystal Materials, Shandong University, Jinan, 250100, China.*

Corresponding Author **E-mail: cgli@email.tjut.edu.cn (C. L.); hu@mail.ipc.ac.cn (Z. H)*

EXPERIMENTAL SECTION

Materials Preparation. The pure-phase polycrystalline powders of the target products, namely $K_2NaLa_2B_3O_9$ (K_2), $K_{2.2}Na_{0.8}La_2B_3O_9$ ($K_{2.2}$), $K_{2.4}Na_{0.6}La_2B_3O_9$ ($K_{2.4}$), $K_{2.6}Na_{0.4}La_2B_3O_9$ ($K_{2.6}$), $K_{2.8}Na_{0.2}La_2B_3O_9$ ($K_{2.8}$), and $K_3La_2B_3O_9$ (K_3), were prepared through the traditional high-temperature solid-state method. The raw materials, K_2CO_3 (Sinopharm, 99.9%), Na_2CO_3 (Sinopharm, 99.9%), La_2O_3 (Sinopharm, 99.9%), and B_2O_3 (Sinopharm, 99.9%), were initially mixed in an agate mortar in a near-stoichiometric ratio to ensure comprehensive intermingling and uniform distribution. Subsequently, the mixture was pre-sintered in a platinum crucible at 450 ℃ for 24 h in a muffle furnace to remove moisture and volatile impurities, while also facilitating initial reactions among the components. Following the pre-sintering, the mixture was sintered at temperatures above 700 ℃ for 48 h, with multiple grinding sessions conducted during this period to ensure complete reactions. Ultimately, the desired target products were successfully synthesized.

Powder X ray Diffraction. The powder X-ray diffraction (XRD) pattern of the target products was recorded using the Rigaku SmartLab 9 kW diffractometer, which employed Kα radiation generated by a single Cu target (λ = 1.5418 Å) as the output light source. During the testing process, the data acquisition range was set from 10° to 70° to cover the potential diffraction peaks associated with the crystal phases of the target product. Furthermore, the acquisition step width was set at 0.02° to achieve a fine scan of the XRD pattern.

Thermal Analysis. The thermal stability and thermal behavior characteristics of the target products were studied using thermogravimetric analysis (TG) and differential scanning calorimetry (DTA) on the NETZSCH STA 449F5 instrument. The experimental procedure involved subjecting the sample to a controlled heating process under a N_2 atmosphere, with the temperature gradually increased from room temperature to 1000 ℃ at a constant rate of 10 ℃/min.

Crystal preparation and structural determination. K_2 and K_3 single crystals were prepared using spontaneous crystallization techniques with B_2O_3-K_2CO_3 as a flux. To characterize their crystal structures, single-crystal XRD analysis was performed. Crystallographic data were collected using the Bruker D8 VENTURE CMOS X-ray equipped with Mo Kα radiation (λ=0.71073 Å). Subsequent data corrections were made using the APEX III software, and crystal models were processed based on the SHELXTL software system, followed by refinement using the direct method.[1] The structural symmetries of K_2 and K_3 were identified utilizing the PLATON program,[2] providing detailed crystallographic information. All relevant data are listed in Table S2 and S3.

Second-harmonic generation (SHG) characterization. Using a Q-switched Nd: YAG solid-state laser operating at a wavelength of 1064 nm, the SHG response of polycrystalline powders was accurately measured following the Kurtz Perry method [3]. During the experiment, both the prepared samples and KDP polycrystalline powders, used as references, underwent a thorough grinding and sieving process to obtain particles with different size ranges: 25-45, 45-63, 63-74, 74-106, 106-150, and 150-210 μm. These particles were pressed into a specialized test container with a diameter of 8 millimeters and 1 millimeter in thickness for testing purposes.

Optical spectroscopy. The UV−vis−NIR diffuse reflectance spectra of the obtained samples were measured at room temperature using a Hitachi UV−vis−NIR spectrophotometer, covering a wavelength range of 200-2000 nm. The experimental bandgap of the samples was determined by analyzing the Kubelka-Munk function.[4] Additionally, the infrared spectra of the samples within the wavelength range of 425-2000 cm^{-1} were recorded using a Nicolet Is50 infrared spectrometer.

Birefringence Properties. The micro-birefringence characteristics of K_2 and K_3 crystals were experimentally assessed using a polarizing microscope (Nikon Eclipse E200MVPOL), and the thickness of the title single crystals

was determined with a Bruker SMART APEX III CCD diffractometer. The calculation of micro-birefringence was conducted based on the formula $R=|Ne–No|\times d = \Delta n \times d$, where Δn, R, and d represent the birefringence,[5] optical path difference, and thickness of the crystals, respectively.

Computational details. A comprehensive investigation of the band structure, partial density of states (PDOS), and total density of states (TDOS) of K_2 and K_3 was conducted utilizing the CASTEP program.[6] Norm-conserving pseudopotentials were employed to accurately model the behavior of the selected valence electrons: B $2s^22p^1$, Na $2s^22p^63s^1$, K $3s^23p^64s^1$, La $5d^16s^2$, and O $2s^22p^4$. To ensure precise calculations, a plane-wave energy cutoff of 700 eV was employed. A detailed sampling and numerical integration were performed using a 4×3×2 Monkhorst-Pack *k*-point grid in the Brillouin zone, providing sufficient sampling in momentum space for accurate band structure. The Perdew-Burke-Ernzerhof (PBE) functional, encompassing the generalized gradient approximation (GGA), was chosen to accurately describe the exchange-correlation energy and adequately account for spatial variations in electron density.[7] This approach significantly enhanced the reliability and accuracy of the computational results.

Table S1 Crystal data and structural refinement parameters of K_2 and K_3.

empirical formula	$K_2NaLa_2B_3O_9$	$K_3La_2B_3O_9$
formula weight	555.44	571.55
temperature	297 K	297 K
crystal system	Orthorhombic	Orthorhombic
space group	*Amm*2	*Pnc*2
a (Å)	5.1775(4)	6.7996(13)
b (Å)	11.4175(10)	16.420(3)
c (Å)	7.2715(5)	9.4868(17)
Z	2	4
V (Å^3)	429.78(6)	1059.2(3)
Density (g/cm^3)	4.292	3.584
F (000)	500	1032
data/restraints/parameters	613/19/56	3015/21/188
R (int)	0.0508	0.0399
GOF (F^2)	1.121	0.983
final R indices [I>2σ (I)][a]	R_1 = 0.0497, wR_2 = 0.1276	R_1 = 0.0399, wR_2 = 0.0938
R indices (all data)	R_1 = 0.0500, wR_2 = 0.1279	R_1 = 0.0518, wR_2 = 0.0960
absolute structure parameter	0.03(10)	0.07(3)
CCDC number	2374500	2374501

[a] $R_1 = \sum||F_o| - |F_c||/\sum|F_o|$; $wR_2 = [\sum w(F_o^2 - F_c^2)^2/\sum w(F_o^2)^2]^{1/2}$.

Table S2. Bond lengths [Å] and angles [°] for K_2.

La(1)-O(1)	2.466(12)	K(3)-O(3)1	3.044(18)
La(1)-O(1)	2.638(15)	K(3)-O(3)16	3.044(18)
La(1)-B(7)	2.92(2)	Na(4)-O(1)5	2.666(19)
La(1)-O(4)	2.450(2)	Na(4)-O(1)0	2.666(19)
La(1)-O(2)3	2.601(10)	Na(4)-B(7)15	3.10(3)
La(1)-O(2)4	2.546(10)	Na(4)-O(4)9	2.80(2)
La(1)-O(2)5	2.601(10)	Na(4)-O(2)	2.301(9)
La(1)-O(2)6	2.546(10)	Na(4)-O(2)17	2.301(9)
La(1)-O(3)6	2.687(5)	Na(4)-O(2)18	2.301(9)
La(1)-O(3)1	2.687(5)	Na(4)-O(2)12	2.301(9)
La(1)-B(3)6	3.017(14)	O(1)-B(7)	1.38(2)
La(1)-B(3)1	3.017(14)	B(7)-O(4)	1.37(4)
K(2)-Na(4)	3.182(10)	O(2)-B(3)	1.379(12)
K(2)-Na(4)7	3.182(10)	O(3)-B(3)	1.36(2)
K(2)-O(4)8	2.759(9)	O(1)-La(1)-O(1)	150.6(5)
K(2)-O(4)9	2.759(9)	O(1)-La(1)-B(7)	28.1(6)
K(2)-O(2)10	2.561(11)	O(1)-La(1)-B(7)	178.7(6)
K(2)-O(2)	2.561(11)	O(1)-La(1)-O(2)4	80.0(4)
K(2)-O(2)11	2.561(11)	O(1)-La(1)-O(2)3	77.5(3)
K(2)-O(2)12	2.561(11)	O(1)-La(1)-O(2)6	80.0(4)
K(2)-O(3)13	2.501(18)	O(1)-La(1)-O(2)5	77.5(3)
K(2)-O(3)14	2.501(18)	O(1)-La(1)-O(3)6	74.5(3)
K(2)-B(3)10	2.91(2)	O(1)-La(1)-O(3)6	103.4(4)
K(2)-B(3)	2.91(2)	O(1)-La(1)-O(3)1	74.5(3)
K(3)-O(1)0	2.893(6)	O(1)-La(1)-O(3)1	103.4(4)
K(3)-O(1)5	2.893(6)	O(1)-La(1)-B(3)6	76.3(5)
K(3)-O(1)	2.893(6)	O(1)-La(1)-B(3)1	76.3(5)
K(3)-O(1)	2.893(6)	O(1)-La(1)-B(3)1	89.0(4)
K(3)-B(7)	2.864(16)	O(1)-La(1)-B(3)6	89.0(4)
K(3)-B(7)15	2.864(16)	B(7)-La(1)-B(3)6	90.3(5)
K(3)-O(2)11	3.171(11)	B(7)-La(1)-B(3)1	90.3(5)
K(3)-O(2)	3.171(11)	O(4)-La(1)-O(1)	153.4(5)
K(3)-O(2)12	3.171(11)	O(4)-La(1)-O(1)	56.0(5)
K(3)-O(2)10	3.171(11)	O(4)-La(1)-B(7)	27.9(7)
La(1)-O(1)	2.466(12)	O(4)-La(1)-O(2)3	80.1(4)
O(4)-La(1)-O(2)5	80.1(4)	O(2)6-La(1)-B(3)1	92.4(5)

O(4)-La(1)-O(2)4	121.5(4)	O(3)1-La(1)-B(7)	76.4(4)
O(4)-La(1)-O(2)6	121.5(4)	O(3)6-La(1)-B(7)	76.4(4)
O(4)-La(1)-O(3)1	81.6(4)	O(3)1-La(1)-O(3)6	148.9(7)
O(4)-La(1)-O(3)6	81.6(4)	O(3)6-La(1)-B(3)1	139.6(6)
O(4)-La(1)-B(3)6	104.2(4)	O(3)6-La(1)-B(3)6	26.9(4)
O(4)-La(1)-B(3)1	104.2(4)	O(3)1-La(1)-B(3)1	26.9(4)
O(2)5-La(1)-O(1)	126.2(3)	O(3)1-La(1)-B(3)6	139.6(6)
O(2)6-La(1)-O(1)	75.4(3)	B(3)1-La(1)-B(3)6	118.2(9)
O(2)3-La(1)-O(1)	126.2(3)	Na(4)7-K(2)-Na(4)	108.9(5)
O(2)4-La(1)-O(1)	75.4(3)	O(4)8-K(2)-Na(4)	164.7(6)
O(2)4-La(1)-B(7)	99.0(5)	O(4)8-K(2)-Na(4)7	55.8(4)
O(2)5-La(1)-B(7)	103.6(5)	O(4)9-K(2)-Na(4)7	164.7(6)
O(2)6-La(1)-B(7)	99.0(5)	O(4)9-K(2)-Na(4)	55.8(4)
O(2)3-La(1)-B(7)	103.6(5)	O(4)9-K(2)-O(4)8	139.6(10)
O(2)4-La(1)-O(2)5	110.2(3)	O(4)9-K(2)-B(3)10	101.2(3)
O(2)6-La(1)-O(2)5	157.4(3)	O(4)9-K(2)-B(3)	101.2(3)
O(2)4-La(1)-O(2)6	65.8(4)	O(4)8-K(2)-B(3)10	101.2(3)
O(2)3-La(1)-O(2)5	64.2(4)	O(4)8-K(2)-B(3)	101.2(3)
O(2)4-La(1)-O(2)3	157.4(3)	O(2)10-K(2)-Na(4)	93.9(4)
O(2)6-La(1)-O(2)3	110.2(3)	O(2)-K(2)-Na(4)7	93.9(4)
O(2)5-La(1)-O(3)1	69.4(4)	O(2)11-K(2)-Na(4)	93.9(4)
O(2)6-La(1)-O(3)6	53.6(4)	O(2)12-K(2)-Na(4)	45.7(2)
O(2)3-La(1)-O(3)1	132.3(4)	O(2)-K(2)-Na(4)	45.7(2)
O(2)4-La(1)-O(3)6	116.9(4)	O(2)10-K(2)-Na(4)7	45.7(2)
O(2)4-La(1)-O(3)1	53.6(4)	O(2)11-K(2)-Na(4)7	45.7(2)
O(2)5-La(1)-O(3)6	132.3(4)	O(2)12-K(2)-Na(4)7	93.9(4)
O(2)3-La(1)-O(3)6	69.4(4)	O(2)11-K(2)-O(4)9	129.0(3)
O(2)6-La(1)-O(3)1	116.9(4)	O(2)11-K(2)-O(4)8	75.3(3)
O(2)5-La(1)-B(3)1	87.7(5)	O(2)12-K(2)-O(4)9	75.3(3)
O(2)4-La(1)-B(3)6	92.4(5)	O(2)10-K(2)-O(4)9	129.0(3)
O(2)4-La(1)-B(3)1	27.0(4)	O(2)-K(2)-O(4)8	129.0(3)
O(2)3-La(1)-B(3)1	150.8(6)	O(2)-K(2)-O(4)9	75.3(3)
O(2)6-La(1)-B(3)6	27.0(4)	O(2)10-K(2)-O(4)8	75.3(3)
O(2)5-La(1)-B(3)6	150.8(6)	O(2)12-K(2)-O(4)8	129.0(3)
O(2)3-La(1)-B(3)6	87.7(5)	O(2)-K(2)-O(2)11	56.2(4)
O(2)10-K(2)-O(2)12	56.2(4)	B(3)10-K(2)-Na(4)7	70.8(3)
O(2)-K(2)-O(2)10	114.2(6)	B(3)-K(2)-Na(4)7	70.8(3)

O(2)-K(2)-O(2)12	88.1(5)	B(3)10-K(2)-B(3)	111.2(11)
O(2)10-K(2)-O(2)11	88.1(5)	O(1)2-K(3)-O(1)15	159.4(7)
O(2)12-K(2)-O(2)11	114.2(6)	O(1)15-K(3)-O(1)	127.0(4)
O(2)10-K(2)-B(3)	105.5(6)	O(1)2-K(3)-O(1)	48.3(4)
O(2)11-K(2)-B(3)	28.3(2)	O(1)15-K(3)-O(1)10	48.3(4)
O(2)12-K(2)-B(3)	105.5(6)	O(1)2-K(3)-O(1)10	127.0(4)
O(2)-K(2)-B(3)	28.3(2)	O(1)10-K(3)-O(1)	159.4(7)
O(2)-K(2)-B(3)10	105.5(6)	O(1)2-K(3)-O(2)	134.5(4)
O(2)10-K(2)-B(3)10	28.3(2)	O(1)10-K(3)-O(2)12	64.0(3)
O(2)12-K(2)-B(3)10	28.3(2)	O(1)-K(3)-O(2)	104.0(3)
O(2)11-K(2)-B(3)10	105.5(6)	O(1)2-K(3)-O(2)12	104.0(3)
O(3)13-K(2)-Na(4)	108.3(2)	O(1)10-K(3)-O(2)11	134.5(4)
O(3)13-K(2)-Na(4)7	108.3(2)	O(1)10-K(3)-O(2)10	104.0(3)
O(3)14-K(2)-Na(4)7	108.3(2)	O(1)15-K(3)-O(2)	64.0(3)
O(3)14-K(2)-Na(4)	108.3(2)	O(1)-K(3)-O(2)11	64.0(3)
O(3)14-K(2)-O(4)8	79.2(3)	O(1)10-K(3)-O(2)	91.2(3)
O(3)14-K(2)-O(4)9	79.2(3)	O(1)15-K(3)-O(2)11	104.0(3)
O(3)13-K(2)-O(4)8	79.2(3)	O(1)2-K(3)-O(2)10	64.0(3)
O(3)13-K(2)-O(4)9	79.2(3)	O(1)-K(3)-O(2)10	91.2(3)
O(3)14-K(2)-O(2)	151.5(2)	O(1)15-K(3)-O(2)10	134.5(4)
O(3)13-K(2)-O(2)12	151.5(2)	O(1)15-K(3)-O(2)12	91.2(3)
O(3)14-K(2)-O(2)10	73.0(4)	O(1)2-K(3)-O(2)11	91.2(3)
O(3)14-K(2)-O(2)11	151.5(2)	O(1)-K(3)-O(2)12	134.5(4)
O(3)13-K(2)-O(2)10	151.5(2)	O(1)2-K(3)-O(3)16	98.9(3)
O(3)13-K(2)-O(2)11	73.0(4)	O(1)10-K(3)-O(3)16	98.9(3)
O(3)14-K(2)-O(2)12	73.0(4)	O(1)15-K(3)-O(3)16	65.7(3)
O(3)13-K(2)-O(2)	73.0(4)	O(1)10-K(3)-O(3)1	65.7(3)
O(3)13-K(2)-O(3)14	114.6(9)	O(1)-K(3)-O(3)1	98.9(3)
O(3)13-K(2)-B(3)10	178.3(8)	O(1)-K(3)-O(3)16	65.7(3)
O(3)13-K(2)-B(3)	67.1(7)	O(1)15-K(3)-O(3)1	98.9(3)
O(3)14-K(2)-B(3)10	67.1(7)	O(1)2-K(3)-O(3)1	65.7(3)
O(3)14-K(2)-B(3)	178.3(8)	B(7)15-K(3)-O(1)10	27.7(4)
B(3)10-K(2)-Na(4)	70.8(3)	B(7)15-K(3)-O(1)15	27.7(4)
B(3)-K(2)-Na(4)	70.8(3)	B(7)-K(3)-O(1)10	137.1(6)
B(7)15-K(3)-O(1)2	137.1(6)	K(2)15-Na(4)-K(3)	179.7(4)
B(7)-K(3)-O(1)	27.7(4)	K(2)-Na(4)-K(3)15	179.7(4)
B(7)-K(3)-O(1)15	137.1(6)	K(3)-Na(4)-K(3)15	108.3(4)

B(7)-K(3)-O(1)2	27.7(4)	O(1)10-Na(4)-K(2)	121.4(2)
B(7)15-K(3)-O(1)	137.1(6)	O(1)10-Na(4)-K(2)15	121.4(2)
B(7)15-K(3)-B(7)	129.4(13)	O(1)15-Na(4)-K(2)15	121.4(2)
B(7)-K(3)-O(2)11	88.3(6)	O(1)15-Na(4)-K(2)	121.4(2)
B(7)15-K(3)-O(2)11	131.1(5)	O(1)10-Na(4)-K(3)	58.3(2)
B(7)15-K(3)-O(2)	88.3(6)	O(1)10-Na(4)-K(3)15	58.3(2)
B(7)-K(3)-O(2)10	88.3(6)	O(1)15-Na(4)-K(3)15	58.3(2)
B(7)15-K(3)-O(2)10	131.1(5)	O(1)15-Na(4)-K(3)	58.3(2)
B(7)-K(3)-O(2)12	131.1(5)	O(1)10-Na(4)-O(1)15	52.7(5)
B(7)15-K(3)-O(2)12	88.3(6)	O(1)10-Na(4)-B(7)15	26.4(2)
B(7)-K(3)-O(2)	131.1(5)	O(1)15-Na(4)-B(7)15	26.4(2)
B(7)15-K(3)-O(3)1	72.0(5)	O(1)10-Na(4)-O(4)9	153.6(2)
B(7)-K(3)-O(3)16	72.0(5)	O(1)15-Na(4)-O(4)9	153.6(2)
B(7)-K(3)-O(3)1	72.0(5)	B(7)15-Na(4)-K(2)15	125.5(3)
B(7)15-K(3)-O(3)16	72.0(5)	B(7)15-Na(4)-K(2)	125.5(3)
O(2)11-K(3)-O(2)12	85.4(4)	B(7)15-Na(4)-K(3)15	54.1(2)
O(2)-K(3)-O(2)12	68.3(4)	B(7)15-Na(4)-K(3)	54.1(2)
O(2)11-K(3)-O(2)10	68.3(4)	O(4)9-Na(4)-K(2)15	54.5(3)
O(2)-K(3)-O(2)11	44.7(3)	O(4)9-Na(4)-K(2)	54.5(3)
O(2)12-K(3)-O(2)10	44.7(3)	O(4)9-Na(4)-K(3)15	125.9(2)
O(2)-K(3)-O(2)10	85.4(4)	O(4)9-Na(4)-K(3)	125.9(2)
O(3)1-K(3)-O(2)12	98.2(3)	O(4)9-Na(4)-B(7)15	180.0
O(3)16-K(3)-O(2)	98.2(3)	O(2)12-Na(4)-K(2)15	111.9(4)
O(3)1-K(3)-O(2)	156.81(18)	O(2)17-Na(4)-K(2)	111.9(4)
O(3)1-K(3)-O(2)11	156.81(18)	O(2)-Na(4)-K(2)15	111.9(4)
O(3)16-K(3)-O(2)10	156.81(18)	O(2)18-Na(4)-K(2)15	52.8(3)
O(3)1-K(3)-O(2)10	98.2(3)	O(2)-Na(4)-K(2)	52.8(3)
O(3)16-K(3)-O(2)11	98.2(3)	O(2)18-Na(4)-K(2)	111.9(4)
O(3)16-K(3)-O(2)12	156.81(18)	O(2)12-Na(4)-K(2)	52.8(3)
O(3)1-K(3)-O(3)16	87.5(7)	O(2)17-Na(4)-K(2)15	52.8(3)
K(2)-Na(4)-K(2)15	108.9(5)	O(2)17-Na(4)-K(3)	127.2(4)
K(2)-Na(4)-K(3)	71.4(2)	O(2)18-Na(4)-K(3)	127.2(4)
K(2)15-Na(4)-K(3)15	71.4(2)	O(2)12-Na(4)-K(3)15	127.2(4)
O(2)-Na(4)-K(3)	68.3(3)	Na(4)7-O(1)-K(3)7	70.0(3)
O(2)17-Na(4)-K(3)15	68.3(3)	B(7)-O(1)-La(1)2	87.5(15)
O(2)12-Na(4)-K(3)	68.3(3)	B(7)-O(1)-La(1)19	178.6(17)
O(2)18-Na(4)-K(3)15	68.3(3)	B(7)-O(1)-K(3)	74.9(6)

O(2)-Na(4)-K(3)15	127.2(4)	B(7)-O(1)-K(3)7	74.9(6)
O(2)-Na(4)-O(1)10	121.5(5)	B(7)-O(1)-Na(4)7	94.5(15)
O(2)17-Na(4)-O(1)15	121.5(5)	La(1)2-B(7)-La(1)	113.0(12)
O(2)12-Na(4)-O(1)15	121.5(5)	La(1)-B(7)-K(2)20	68.4(5)
O(2)17-Na(4)-O(1)10	80.5(4)	La(1)-B(7)-K(2)21	68.4(5)
O(2)-Na(4)-O(1)15	80.5(4)	La(1)2-B(7)-K(2)20	68.4(5)
O(2)18-Na(4)-O(1)15	80.5(4)	La(1)2-B(7)-K(2)21	68.4(5)
O(2)12-Na(4)-O(1)10	80.5(4)	La(1)2-B(7)-Na(4)7	123.5(6)
O(2)18-Na(4)-O(1)10	121.5(5)	La(1)-B(7)-Na(4)7	123.5(6)
O(2)18-Na(4)-B(7)15	101.5(4)	K(2)21-B(7)-K(2)20	96.1(9)
O(2)-Na(4)-B(7)15	101.5(4)	K(3)-B(7)-La(1)	103.65(14)
O(2)17-Na(4)-B(7)15	101.5(4)	K(3)7-B(7)-La(1)	103.65(14)
O(2)12-Na(4)-B(7)15	101.5(4)	K(3)7-B(7)-La(1)2	103.65(14)
O(2)17-Na(4)-O(4)9	78.5(4)	K(3)-B(7)-La(1)2	103.65(14)
O(2)18-Na(4)-O(4)9	78.5(4)	K(3)-B(7)-K(2)21	163.4(11)
O(2)12-Na(4)-O(4)9	78.5(4)	K(3)-B(7)-K(2)20	67.2(3)
O(2)-Na(4)-O(4)9	78.5(4)	K(3)7-B(7)-K(2)21	67.2(3)
O(2)18-Na(4)-O(2)12	157.0(9)	K(3)7-B(7)-K(2)20	163.4(11)
O(2)-Na(4)-O(2)12	101.4(5)	K(3)-B(7)-K(3)7	129.4(13)
O(2)12-Na(4)-O(2)17	73.9(4)	K(3)-B(7)-Na(4)7	64.7(7)
O(2)-Na(4)-O(2)18	73.9(4)	K(3)7-B(7)-Na(4)7	64.7(7)
O(2)-Na(4)-O(2)17	157.0(9)	Na(4)7-B(7)-K(2)20	131.9(5)
O(2)18-Na(4)-O(2)17	101.4(5)	Na(4)7-B(7)-K(2)21	131.9(5)
La(1)19-O(1)-La(1)2	93.9(4)	O(1)-B(7)-La(1)2	64.4(10)
La(1)19-O(1)-K(3)	104.5(3)	O(1)-B(7)-La(1)	177(2)
La(1)2-O(1)-K(3)	110.6(3)	O(1)2-B(7)-La(1)2	177(2)
La(1)2-O(1)-K(3)7	110.6(3)	O(1)2-B(7)-La(1)	64.4(10)
La(1)19-O(1)-K(3)7	104.5(3)	O(1)2-B(7)-K(2)21	110.0(7)
La(1)19-O(1)-Na(4)7	84.2(5)	O(1)2-B(7)-K(2)20	110.0(7)
La(1)2-O(1)-Na(4)7	178.0(5)	O(1)-B(7)-K(2)20	110.0(7)
K(3)7-O(1)-K(3)	126.9(4)	O(1)-B(7)-K(2)21	110.0(7)
Na(4)7-O(1)-K(3)	70.0(3)	O(1)2-B(7)-K(3)7	77.3(9)
O(1)-B(7)-K(3)7	77.3(9)	Na(4)-O(2)-La(1)23	92.1(4)
O(1)-B(7)-K(3)	77.3(9)	Na(4)-O(2)-La(1)22	90.3(4)
O(1)2-B(7)-K(3)	77.3(9)	Na(4)-O(2)-K(2)	81.6(4)
O(1)-B(7)-Na(4)7	59.2(15)	Na(4)-O(2)-K(3)	69.4(3)
O(1)2-B(7)-Na(4)7	59.2(15)	B(3)-O(2)-La(1)22	95.9(10)

O(1)2-B(7)-O(1)	118(3)	B(3)-O(2)-La(1)23	119.9(12)
O(4)-B(7)-La(1)	56.5(6)	B(3)-O(2)-K(2)	90.0(10)
O(4)-B(7)-La(1)2	56.5(6)	B(3)-O(2)-K(3)	77.6(10)
O(4)-B(7)-K(2)21	48.1(5)	B(3)-O(2)-Na(4)	146.8(11)
O(4)-B(7)-K(2)20	48.1(5)	La(1)19-O(3)-La(1)22	148.9(7)
O(4)-B(7)-K(3)	115.3(7)	La(1)19-O(3)-K(3)19	104.9(4)
O(4)-B(7)-K(3)7	115.3(7)	La(1)22-O(3)-K(3)19	104.9(4)
O(4)-B(7)-Na(4)7	180.0	K(2)24-O(3)-La(1)22	88.6(4)
O(4)-B(7)-O(1)2	120.8(15)	K(2)24-O(3)-La(1)19	88.6(4)
O(4)-B(7)-O(1)	120.8(15)	K(2)24-O(3)-K(3)19	78.9(5)
La(1)2-O(4)-La(1)	168.7(9)	B(3)-O(3)-La(1)22	90.2(5)
La(1)2-O(4)-K(2)20	88.06(19)	B(3)-O(3)-La(1)19	90.2(5)
La(1)-O(4)-K(2)21	88.06(19)	B(3)-O(3)-K(2)24	175.4(16)
La(1)2-O(4)-K(2)21	88.06(19)	B(3)-O(3)-K(3)19	105.6(14)
La(1)-O(4)-K(2)20	88.06(19)	La(1)19-B(3)-La(1)22	118.2(9)
La(1)2-O(4)-Na(4)21	84.4(4)	La(1)22-B(3)-K(3)	86.5(6)
La(1)-O(4)-Na(4)21	84.4(4)	La(1)19-B(3)-K(3)	86.5(6)
K(2)21-O(4)-K(2)20	139.6(10)	K(2)-B(3)-La(1)22	118.6(3)
K(2)20-O(4)-Na(4)21	69.8(5)	K(2)-B(3)-La(1)19	118.6(3)
K(2)21-O(4)-Na(4)21	69.8(5)	K(2)-B(3)-K(3)	75.3(4)
B(7)-O(4)-La(1)	95.6(4)	O(2)11-B(3)-La(1)22	163.2(16)
B(7)-O(4)-La(1)2	95.6(4)	O(2)11-B(3)-La(1)19	57.1(7)
B(7)-O(4)-K(2)20	110.2(5)	O(2)-B(3)-La(1)22	57.1(7)
B(7)-O(4)-K(2)21	110.2(5)	O(2)-B(3)-La(1)19	163.2(16)
B(7)-O(4)-Na(4)21	180.000(11)	O(2)11-B(3)-K(2)	61.7(9)
La(1)22-O(2)-La(1)23	92.9(3)	O(2)-B(3)-K(2)	61.7(9)
La(1)22-O(2)-K(2)	171.7(4)	O(2)11-B(3)-K(3)	77.3(9)
La(1)23-O(2)-K(3)	159.8(3)	O(2)-B(3)-K(3)	77.3(9)
La(1)22-O(2)-K(3)	95.3(3)	O(2)11-B(3)-O(2)	122.0(16)
K(2)-O(2)-La(1)23	89.3(4)	O(3)-B(3)-La(1)22	62.9(6)
K(2)-O(2)-K(3)	80.2(3)	O(3)-B(3)-La(1)19	62.9(6)
O(3)-B(3)-K(2)	173.7(18)	O(3)-B(3)-O(2)	118.8(9)
O(3)-B(3)-K(3)	111.0(17)	O(3)-B(3)-O(2)11	118.8(9)

Symmetry transformations used to generate equivalent atoms:

#1 x,y-1/2,z-1/2 #2 -x-2,-y-1,z #3 x-1,-y-1,z-1

#4 -x-1,y-1/2,z-1/2 #5 -x-1,-y-1,z-1 #6 x-1,y-1/2,z-1/2

#7 x-1,y,z #8 x,y,z+1 #9 x+1,y,z+1 #10 -x-1,-y-1,z

#11 -x-1,y,z #12 x,-y-1,z #13 -x-1,-y-1/2,z+1/2

#14 x,y-1/2,z+1/2 #15 x+1,y,z #16 -x-1,-y-1/2,z-1/2

#17 -x,-y-1,z #18 -x,y,z #19 x,y+1/2,z+1/2

#20 x,y,z-1 #21 x-1,y,z-1 #22 x+1,y+1/2,z+1/2

#23 -x-1,-y-1,z+1 #24 x,y+1/2,z-1/2

Table S3. Bond lengths [Å] and angles [°] for K_3

La(1)-La(3)1	3.511(3)	K(1)-O(3)	2.80(3)
La(1)-K(2)2	3.358(4)	K(1)-O(3)10	2.779(16)
La(1)-O(1)	2.874(9)	K(1)-O(4)	2.944(10)
La(1)-O(2)3	2.86(3)	K(1)-O(4)6	2.791(10)
La(1)-O(2)	2.85(2)	K(1)-O(5)9	3.25(3)
La(1)-O(3)4	3.02(2)	K(1)-O(5)4	2.45(2)
La(1)-O(6)1	2.744(9)	K(1)-O(8)	2.746(9)
La(1)-O(7)4	2.734(2)	K(1)-O(8)6	2.786(9)
La(1)-O(8)4	2.722(9)	K(1)-O(10)10	2.933(10)
La(1)-O(11)	2.570(10)	K(1)-B(1)10	3.184(13)
La(1)-B(4)4	3.021(7)	K(1)-B(4)	3.019(14)
La(1)-O(3A)4	2.57(6)	K(1)-O(3A)10	3.16(7)
La(2)-O(1)	2.539(9)	K(2)-O(1)	2.793(10)
La(2)-O(1)1	2.558(9)	K(2)-O(2)11	2.64(2)
La(2)-O(3)	2.592(15)	K(2)-O(6)1	2.802(10)
La(2)-O(4)	2.563(8)	K(2)-O(7)4	2.896(3)
La(2)-O(6)1	2.598(8)	K(2)-O(8)4	2.915(9)
La(2)-O(8)	2.412(8)	K(2)-O(9)5	3.26(3)
La(2)-O(10)5	2.433(11)	K(2)-O(10)5	3.075(14)
La(2)-O(11)	2.553(10)	K(2)-O(10)8	3.101(17)
La(2)-B(1)	3.005(13)	K(2)-O(11)5	3.011(10)
La(2)-B(3)	2.957(13)	K(2)-B(4)4	3.140(7)
La(2)-B(3)1	2.942(13)	K(2)-O(3A)8	3.03(7)
La(2)-O(3A)	2.76(5)	K(3)-O(2)3	3.02(3)
La(3)-La(3)6	0.586(3)	K(3)-O(5)3	2.73(2)
La(3)-O(2)4	2.65(3)	K(3)-O(5)	2.91(2)
La(3)-O(4)	2.526(8)	K(3)-O(9)	2.78(2)
La(3)-O(4)6	2.564(8)	K(3)-O(10)	3.156(13)
La(3)-O(5)4	2.65(3)	K(3)-O(10)1	3.299(16)
La(3)-O(6)	2.531(8)	K(3)-O(11)	2.746(11)
La(3)-O(6)6	2.587(8)	K(3)-O(11)1	2.987(12)
La(3)-O(7)7	2.376(10)	K(3)-B(1)	2.949(17)
La(3)-O(9)8	2.45(2)	K(3)-B(1)1	2.96(2)
La(3)-O(9)9	1.87(2)	K(3)-B(2)	2.942(11)
La(3)-B(3)6	2.984(14)	K(3)-O(3A)	3.25(8)
La(3)-B(3)	2.927(13)	O(1)-B(3)	1.362(16)

O(2)-O(9)3	1.12(4)	O(6)1-La(1)-O(2)3	102.2(6)
O(2)-B(2)	1.36(3)	O(6)1-La(1)-O(3)4	154.4(3)
O(3)-B(1)	1.40(2)	O(6)1-La(1)-B(4)4	93.3(3)
O(4)-B(3)	1.409(15)	O(7)4-La(1)-La(3)1	42.5(2)
O(5)-O(5)3	1.40(5)	O(7)4-La(1)-K(2)2	147.24(9)
O(5)-O(9)3	1.39(4)	O(7)4-La(1)-O(1)	101.4(2)
O(5)-B(2)	1.38(3)	O(7)4-La(1)-O(2)	64.8(5)
O(6)-B(3)	1.370(16)	O(7)4-La(1)-O(2)3	102.9(4)
O(7)-B(4)	1.36(2)	O(7)4-La(1)-O(3)4	107.3(5)
O(8)-B(4)	1.352(12)	O(7)4-La(1)-O(6)1	71.9(3)
O(9)-B(2)	1.32(2)	O(7)4-La(1)-B(4)4	26.7(4)
O(10)-B(1)	1.366(18)	O(8)4-La(1)-La(3)1	92.75(19)
O(11)-B(1)	1.368(19)	O(8)4-La(1)-K(2)2	131.4(2)
B(1)-O(3A)	1.30(5)	O(8)4-La(1)-O(1)	72.0(3)
K(2)2-La(1)-La(3)1	125.88(8)	O(8)4-La(1)-O(2)	105.1(5)
O(1)-La(1)-La(3)1	112.9(2)	O(8)4-La(1)-O(2)3	134.2(5)
O(1)-La(1)-K(2)2	110.06(19)	O(8)4-La(1)-O(3)4	62.6(5)
O(1)-La(1)-O(3)4	81.6(3)	O(8)4-La(1)-O(6)1	102.5(3)
O(1)-La(1)-B(4)4	92.9(2)	O(8)4-La(1)-O(7)4	51.0(3)
O(2)-La(1)-La(3)1	47.9(7)	O(8)4-La(1)-B(4)4	26.6(3)
O(2)3-La(1)-La(3)1	78.1(5)	O(11)-La(1)-La(3)1	108.5(3)
O(2)3-La(1)-K(2)2	49.5(4)	O(11)-La(1)-K(2)2	59.3(2)
O(2)-La(1)-K(2)2	86.3(5)	O(11)-La(1)-O(1)	69.5(3)
O(2)3-La(1)-O(1)	152.6(5)	O(11)-La(1)-O(2)	113.6(5)
O(2)-La(1)-O(1)	160.8(7)	O(11)-La(1)-O(2)3	83.3(5)
O(2)-La(1)-O(2)3	38.2(8)	O(11)-La(1)-O(3)4	104.3(5)
O(2)3-La(1)-O(3)4	102.8(6)	O(11)-La(1)-O(6)1	73.5(3)
O(2)-La(1)-O(3)4	114.5(7)	O(11)-La(1)-O(7)4	145.3(3)
O(2)3-La(1)-B(4)4	114.5(5)	O(11)-La(1)-O(8)4	140.8(3)
O(2)-La(1)-B(4)4	80.0(5)	O(11)-La(1)-B(4)4	160.2(2)
O(3)4-La(1)-La(3)1	147.0(4)	O(11)-La(1)-O(3A)4	91.3(19)
O(3)4-La(1)-K(2)2	69.6(4)	B(4)4-La(1)-La(3)1	69.2(3)
O(3)4-La(1)-B(4)4	81.1(5)	B(4)4-La(1)-K(2)2	138.81(19)
O(6)1-La(1)-La(3)1	45.73(17)	O(3A)4-La(1)-O(1)	78.7(12)
O(6)1-La(1)-K(2)2	125.2(2)	O(3A)4-La(1)-O(2)	119.3(14)
O(6)1-La(1)-O(1)	73.7(3)	O(1)-La(2)-O(1)1	137.36(6)
O(6)1-La(1)-O(2)	88.8(7)	O(1)1-La(2)-O(3)	96.7(7)

O(1)-La(2)-O(3)	107.9(5)	O(8)-La(2)-B(3)1	109.1(3)
O(1)-La(2)-O(4)	55.8(3)	O(8)-La(2)-O(3A)	76.0(11)
O(1)1-La(2)-O(4)	160.7(3)	O(10)5-La(2)-O(1)1	84.5(4)
O(1)-La(2)-O(6)1	82.0(3)	O(10)5-La(2)-O(1)	88.8(4)
O(1)1-La(2)-O(6)1	55.3(3)	O(10)5-La(2)-O(3)	152.0(4)
O(1)-La(2)-O(11)	75.4(3)	O(10)5-La(2)-O(4)	81.5(4)
O(1)-La(2)-B(1)	93.5(5)	O(10)5-La(2)-O(6)1	80.6(3)
O(1)1-La(2)-B(1)	92.8(5)	O(10)5-La(2)-O(11)	153.5(3)
O(1)1-La(2)-B(3)1	27.6(3)	O(10)5-La(2)-B(1)	177.3(6)
O(1)-La(2)-B(3)	27.4(3)	O(10)5-La(2)-B(3)	86.0(5)
O(1)1-La(2)-B(3)	162.4(3)	O(10)5-La(2)-B(3)1	81.7(4)
O(1)-La(2)-B(3)1	109.8(3)	O(10)5-La(2)-O(3A)	152.5(12)
O(1)1-La(2)-O(3A)	81.1(17)	O(11)-La(2)-O(1)1	92.8(3)
O(1)-La(2)-O(3A)	117.4(13)	O(11)-La(2)-O(3)	54.5(4)
O(3)-La(2)-O(6)1	122.9(6)	O(11)-La(2)-O(4)	105.7(4)
O(3)-La(2)-B(1)	27.7(5)	O(11)-La(2)-O(6)1	76.3(3)
O(3)-La(2)-B(3)1	111.7(7)	O(11)-La(2)-B(1)	27.0(4)
O(3)-La(2)-B(3)	98.6(7)	O(11)-La(2)-B(3)	89.0(4)
O(4)-La(2)-O(3)	89.6(7)	O(11)-La(2)-B(3)1	83.8(4)
O(4)-La(2)-O(6)1	134.2(3)	O(11)-La(2)-O(3A)	51.1(11)
O(4)-La(2)-B(1)	101.0(4)	B(3)-La(2)-B(1)	96.7(5)
O(4)-La(2)-B(3)1	158.0(4)	B(3)1-La(2)-B(1)	96.1(4)
O(4)-La(2)-B(3)	28.4(3)	B(3)1-La(2)-B(3)	135.8(3)
O(4)-La(2)-O(3A)	105.9(18)	O(3A)-La(2)-B(1)	25.7(11)
O(6)1-La(2)-B(1)	98.3(3)	O(3A)-La(2)-B(3)	113.2(17)
O(6)1-La(2)-B(3)	108.4(3)	La(3)6-La(3)-O(2)4	153.4(6)
O(6)1-La(2)-B(3)1	27.7(3)	La(3)6-La(3)-O(4)6	79.7(4)
O(6)1-La(2)-O(3A)	109.3(17)	La(3)6-La(3)-O(4)	87.1(4)
O(8)-La(2)-O(1)1	82.9(3)	La(3)6-La(3)-O(5)4	155.3(5)
O(8)-La(2)-O(1)	136.9(3)	La(3)6-La(3)-O(6)	88.9(5)
O(8)-La(2)-O(3)	73.3(4)	La(3)6-La(3)-O(6)6	78.0(5)
O(8)-La(2)-O(4)	81.5(3)	La(3)6-La(3)-O(7)7	82.92(5)
O(8)-La(2)-O(6)1	134.9(3)	La(3)6-La(3)-O(9)9	170.7(10)
O(8)-La(2)-O(10)5	79.2(4)	La(3)6-La(3)-O(9)8	7.1(7)
O(8)-La(2)-O(11)	126.8(3)	La(3)6-La(3)-B(3)	89.9(5)
O(8)-La(2)-B(1)	100.0(4)	La(3)6-La(3)-B(3)6	78.8(5)
O(8)-La(2)-B(3)	109.9(3)	O(8)-La(2)-B(3)1	109.1(3)

O(2)4-La(3)-B(3)	108.1(6)	O(9)8-La(3)-O(2)4	152.7(10)
O(2)4-La(3)-B(3)6	98.1(6)	O(9)9-La(3)-O(4)	101.2(9)
O(4)6-La(3)-O(2)4	110.0(6)	O(9)8-La(3)-O(4)6	86.1(7)
O(4)-La(3)-O(2)4	118.1(6)	O(9)9-La(3)-O(4)6	97.2(8)
O(4)-La(3)-O(4)6	82.9(4)	O(9)8-La(3)-O(4)	84.9(7)
O(4)6-La(3)-O(5)4	82.8(6)	O(9)8-La(3)-O(5)4	156.6(8)
O(4)-La(3)-O(5)4	73.4(6)	O(9)9-La(3)-O(5)4	30.0(10)
O(4)-La(3)-O(6)	56.5(3)	O(9)8-La(3)-O(6)6	84.1(6)
O(4)6-La(3)-O(6)6	55.4(3)	O(9)8-La(3)-O(6)	82.1(6)
O(4)-La(3)-O(6)6	137.3(3)	O(9)9-La(3)-O(6)	99.1(8)
O(4)-La(3)-B(3)6	110.8(3)	O(9)9-La(3)-O(6)6	93.0(8)
O(4)6-La(3)-B(3)	111.5(3)	O(9)9-La(3)-O(7)7	93.4(9)
O(4)-La(3)-B(3)	28.8(3)	O(9)9-La(3)-O(9)8	173.4(15)
O(4)6-La(3)-B(3)6	28.1(3)	O(9)8-La(3)-B(3)6	85.8(7)
O(5)4-La(3)-O(2)4	50.5(8)	O(9)9-La(3)-B(3)	99.3(9)
O(5)4-La(3)-B(3)	80.4(6)	O(9)8-La(3)-B(3)	84.7(6)
O(5)4-La(3)-B(3)6	93.7(6)	O(9)9-La(3)-B(3)6	94.3(8)
O(6)6-La(3)-O(2)4	87.3(5)	B(3)-La(3)-B(3)6	139.2(5)
O(6)-La(3)-O(2)4	98.0(5)	O(3)10-K(1)-O(3)	95.4(4)
O(6)-La(3)-O(4)6	138.4(3)	O(3)10-K(1)-O(4)6	113.8(4)
O(6)6-La(3)-O(5)4	105.9(6)	O(3)-K(1)-O(4)	78.4(4)
O(6)-La(3)-O(5)4	92.8(6)	O(3)10-K(1)-O(4)	163.1(6)
O(6)-La(3)-O(6)6	159.2(3)	O(3)10-K(1)-O(5)9	75.5(7)
O(6)-La(3)-B(3)6	163.1(4)	O(3)-K(1)-O(5)9	79.1(6)
O(6)6-La(3)-B(3)	163.3(4)	O(3)10-K(1)-O(8)6	100.1(6)
O(6)6-La(3)-B(3)6	27.3(3)	O(3)-K(1)-O(10)10	139.8(5)
O(6)-La(3)-B(3)	27.9(3)	O(3)10-K(1)-O(10)10	49.8(5)
O(7)7-La(3)-O(2)4	72.8(6)	O(3)-K(1)-B(1)10	121.2(4)
O(7)7-La(3)-O(4)	137.3(2)	O(3)10-K(1)-B(1)10	26.0(4)
O(7)7-La(3)-O(4)6	135.1(2)	O(3)-K(1)-B(4)	84.7(4)
O(7)7-La(3)-O(5)4	121.8(5)	O(3)10-K(1)-B(4)	104.7(6)
O(7)7-La(3)-O(6)	81.8(2)	O(3)-K(1)-O(3A)10	99.1(10)
O(7)7-La(3)-O(6)6	80.6(2)	O(3)10-K(1)-O(3A)10	13.1(10)
O(7)7-La(3)-O(9)8	80.2(7)	O(4)6-K(1)-O(3)	150.2(4)
O(7)7-La(3)-B(3)	109.6(3)	O(4)6-K(1)-O(4)	71.9(3)
O(7)7-La(3)-B(3)6	107.7(3)	O(4)6-K(1)-O(5)9	101.4(4)
O(9)9-La(3)-O(2)4	20.7(10)	O(4)-K(1)-O(5)9	87.8(4)

O(4)6-K(1)-O(10)10	69.4(3)	O(10)10-K(1)-O(5)9	105.2(5)
O(4)-K(1)-B(1)10	157.3(4)	O(10)10-K(1)-B(1)10	25.4(3)
O(4)6-K(1)-B(1)10	88.4(4)	O(10)10-K(1)-B(4)	86.2(3)
O(4)-K(1)-B(4)	90.5(3)	O(10)10-K(1)-O(3A)10	42.3(11)
O(4)6-K(1)-B(4)	93.4(3)	B(1)10-K(1)-O(5)9	85.3(5)
O(4)-K(1)-O(3A)10	175.9(14)	B(4)-K(1)-O(5)9	163.7(4)
O(4)6-K(1)-O(3A)10	110.7(10)	B(4)-K(1)-B(1)10	102.0(4)
O(5)4-K(1)-O(3)10	95.2(8)	B(4)-K(1)-O(3A)10	92.5(13)
O(5)4-K(1)-O(3)	89.6(7)	O(1)-K(2)-La(1)5	147.0(2)
O(5)4-K(1)-O(4)	69.3(6)	O(1)-K(2)-O(6)1	74.1(3)
O(5)4-K(1)-O(4)6	81.9(7)	O(1)-K(2)-O(7)4	99.4(2)
O(5)4-K(1)-O(5)9	23.5(8)	O(1)-K(2)-O(8)4	70.4(3)
O(5)4-K(1)-O(8)6	152.6(7)	O(1)-K(2)-O(9)5	138.4(5)
O(5)4-K(1)-O(8)	135.1(6)	O(1)-K(2)-O(10)8	69.2(3)
O(5)4-K(1)-O(10)10	110.2(6)	O(1)-K(2)-O(10)5	72.6(3)
O(5)4-K(1)-B(1)10	97.6(7)	O(1)-K(2)-O(11)5	111.5(3)
O(5)4-K(1)-B(4)	159.7(6)	O(1)-K(2)-B(4)4	91.9(2)
O(5)4-K(1)-O(3A)10	107.7(14)	O(1)-K(2)-O(3A)8	109.3(10)
O(8)-K(1)-O(3)	65.3(4)	O(2)11-K(2)-La(1)5	55.4(7)
O(8)6-K(1)-O(3)	111.2(4)	O(2)11-K(2)-O(1)	156.2(8)
O(8)-K(1)-O(3)10	122.4(5)	O(2)11-K(2)-O(6)1	83.1(8)
O(8)6-K(1)-O(4)	96.9(3)	O(2)11-K(2)-O(7)4	65.2(6)
O(8)-K(1)-O(4)6	101.5(3)	O(2)11-K(2)-O(8)4	106.2(6)
O(8)6-K(1)-O(4)6	71.2(3)	O(2)11-K(2)-O(9)5	18.3(8)
O(8)-K(1)-O(4)	69.5(2)	O(2)11-K(2)-O(10)5	105.0(6)
O(8)6-K(1)-O(5)9	169.3(5)	O(2)11-K(2)-O(10)8	131.1(8)
O(8)-K(1)-O(5)9	140.5(5)	O(2)11-K(2)-O(11)5	79.3(6)
O(8)-K(1)-O(8)6	50.0(3)	O(2)11-K(2)-B(4)4	81.0(5)
O(8)-K(1)-O(10)10	112.8(3)	O(2)11-K(2)-O(3A)8	93.8(12)
O(8)6-K(1)-O(10)10	65.3(3)	O(6)1-K(2)-La(1)5	128.6(2)
O(8)6-K(1)-B(1)10	86.8(4)	O(6)1-K(2)-O(7)4	68.7(3)
O(8)-K(1)-B(1)10	127.0(5)	O(6)1-K(2)-O(8)4	96.4(3)
O(8)-K(1)-B(4)	26.59(18)	O(6)1-K(2)-O(9)5	64.8(5)
O(8)6-K(1)-B(4)	26.55(18)	O(6)1-K(2)-O(10)5	67.1(3)
O(8)6-K(1)-O(3A)10	87.0(14)	O(6)1-K(2)-O(10)8	141.8(3)
O(8)-K(1)-O(3A)10	112.4(11)	O(6)1-K(2)-O(11)5	101.3(3)
O(10)10-K(1)-O(4)	140.8(3)	O(6)1-K(2)-B(4)4	89.7(3)

O(6)1-K(2)-O(3A)8	175.6(12)	O(5)3-K(3)-O(9)	29.2(8)
O(7)4-K(2)-La(1)5	110.83(9)	O(5)3-K(3)-O(10)1	93.9(5)
O(7)4-K(2)-O(8)4	47.7(2)	O(5)-K(3)-O(10)1	104.6(6)
O(7)4-K(2)-O(9)5	60.4(4)	O(5)3-K(3)-O(10)	133.6(7)
O(7)4-K(2)-O(10)8	106.9(3)	O(5)-K(3)-O(10)	147.5(5)
O(7)4-K(2)-O(10)5	135.6(3)	O(5)3-K(3)-O(11)1	120.7(6)
O(7)4-K(2)-O(11)5	143.7(3)	O(5)-K(3)-O(11)1	110.5(5)
O(7)4-K(2)-B(4)4	25.6(4)	O(5)3-K(3)-O(11)	121.5(6)
O(7)4-K(2)-O(3A)8	112.8(14)	O(5)3-K(3)-B(1)1	97.2(6)
O(8)4-K(2)-La(1)5	121.6(2)	O(5)3-K(3)-B(1)	143.6(7)
O(8)4-K(2)-O(9)5	107.0(4)	O(5)-K(3)-B(1)	138.0(7)
O(8)4-K(2)-O(10)8	61.6(3)	O(5)-K(3)-B(1)1	96.1(6)
O(8)4-K(2)-O(10)5	142.4(3)	O(5)3-K(3)-B(2)	27.9(6)
O(8)4-K(2)-O(11)5	162.1(3)	O(5)-K(3)-B(2)	27.4(6)
O(8)4-K(2)-B(4)4	25.5(3)	O(5)-K(3)-O(3A)	145.2(11)
O(8)4-K(2)-O(3A)8	87.4(13)	O(5)3-K(3)-O(3A)	165.6(12)
O(9)5-K(2)-La(1)5	71.3(4)	O(9)-K(3)-O(2)3	21.7(7)
O(10)8-K(2)-La(1)5	89.1(2)	O(9)-K(3)-O(5)	48.6(6)
O(10)5-K(2)-La(1)5	93.4(2)	O(9)-K(3)-O(10)	105.2(6)
O(10)5-K(2)-O(9)5	96.3(6)	O(9)-K(3)-O(10)1	111.6(7)
O(10)8-K(2)-O(9)5	148.1(5)	O(9)-K(3)-O(11)1	148.7(7)
O(10)5-K(2)-O(10)8	110.2(2)	O(9)-K(3)-B(1)1	122.9(7)
O(10)5-K(2)-B(4)4	154.7(3)	O(9)-K(3)-B(1)	115.5(8)
O(10)8-K(2)-B(4)4	81.3(4)	O(9)-K(3)-B(2)	26.4(5)
O(11)5-K(2)-La(1)5	47.20(19)	O(9)-K(3)-O(3A)	139.0(13)
O(11)5-K(2)-O(9)5	83.6(4)	O(10)-K(3)-O(10)1	103.4(3)
O(11)5-K(2)-O(10)8	101.6(3)	O(10)-K(3)-O(3A)	40.3(10)
O(11)5-K(2)-O(10)5	46.3(3)	O(11)1-K(3)-O(2)3	164.3(5)
O(11)5-K(2)-B(4)4	156.1(2)	O(11)-K(3)-O(2)3	77.6(6)
O(11)5-K(2)-O(3A)8	75.1(14)	O(11)-K(3)-O(5)	110.5(6)
B(4)4-K(2)-La(1)5	109.64(10)	O(11)-K(3)-O(9)	98.6(7)
B(4)4-K(2)-O(9)5	81.9(5)	O(11)-K(3)-O(10)1	143.7(3)
O(2)3-K(3)-O(10)	94.7(5)	O(11)1-K(3)-O(10)	100.9(4)
O(2)3-K(3)-O(10)1	133.2(7)	O(11)-K(3)-O(10)	47.2(3)
O(2)3-K(3)-O(3A)	120.4(13)	O(11)1-K(3)-O(10)1	44.4(3)
O(5)3-K(3)-O(2)3	45.9(8)	O(11)-K(3)-O(11)1	111.9(2)
O(5)3-K(3)-O(5)	28.5(9)	O(11)-K(3)-B(1)1	138.4(4)

O(11)-K(3)-B(1)	27.5(4)	La(3)1-O(2)-K(3)3	88.8(9)
O(11)-K(3)-B(2)	94.3(5)	K(2)11-O(2)-La(1)	105.4(9)
O(11)-K(3)-O(3A)	44.1(11)	K(2)11-O(2)-La(1)3	75.1(8)
O(11)1-K(3)-O(3A)	72.1(11)	K(2)11-O(2)-La(3)1	89.0(7)
B(1)1-K(3)-O(2)3	143.0(7)	K(2)11-O(2)-K(3)3	84.6(6)
B(1)-K(3)-O(2)3	97.8(7)	O(9)3-O(2)-La(1)3	148(2)
B(1)-K(3)-O(10)	25.6(3)	O(9)3-O(2)-La(1)	97.7(19)
B(1)1-K(3)-O(10)1	24.4(3)	O(9)3-O(2)-La(3)1	36.2(15)
B(1)-K(3)-O(10)1	116.8(5)	O(9)3-O(2)-K(2)11	114(2)
B(1)1-K(3)-O(10)	116.1(4)	O(9)3-O(2)-K(3)3	66.7(18)
B(1)1-K(3)-O(11)1	26.6(3)	O(9)3-O(2)-B(2)	63.0(19)
B(1)-K(3)-O(11)1	95.5(5)	B(2)-O(2)-La(1)3	96.6(14)
B(1)-K(3)-B(1)1	118.9(3)	B(2)-O(2)-La(1)	97.2(11)
B(1)-K(3)-O(3A)	23.6(12)	B(2)-O(2)-La(3)1	96.2(18)
B(1)1-K(3)-O(3A)	96.7(12)	B(2)-O(2)-K(2)11	157.4(15)
B(2)-K(3)-O(2)3	26.4(6)	B(2)-O(2)-K(3)3	73.6(12)
B(2)-K(3)-O(10)	120.8(5)	La(1)1-O(3)-K(3)4	169.6(5)
B(2)-K(3)-O(10)1	121.7(5)	La(2)-O(3)-La(1)1	81.3(5)
B(2)-K(3)-O(11)1	137.9(5)	La(2)-O(3)-K(1)	87.9(7)
B(2)-K(3)-B(1)	119.4(8)	La(2)-O(3)-K(1)10	152.3(12)
B(2)-K(3)-B(1)1	120.7(6)	La(2)-O(3)-K(3)4	89.8(6)
B(2)-K(3)-O(3A)	138.1(11)	K(1)10-O(3)-La(1)1	120.2(6)
La(2)4-O(1)-La(1)	84.7(3)	K(1)-O(3)-La(1)1	96.1(4)
La(2)-O(1)-La(1)	87.8(3)	K(1)10-O(3)-K(1)	73.5(6)
La(2)-O(1)-La(2)4	172.0(4)	K(1)10-O(3)-K(3)4	69.9(5)
La(2)-O(1)-K(2)	89.0(3)	K(1)-O(3)-K(3)4	88.9(8)
La(2)4-O(1)-K(2)	92.3(3)	B(1)-O(3)-La(1)1	114.1(18)
K(2)-O(1)-La(1)	77.5(3)	B(1)-O(3)-La(2)	92.8(9)
B(3)-O(1)-La(1)	157.5(8)	B(1)-O(3)-K(1)	149.6(19)
B(3)-O(1)-La(2)	93.7(7)	B(1)-O(3)-K(1)10	93.3(9)
B(3)-O(1)-La(2)4	92.1(8)	B(1)-O(3)-K(3)4	60.7(13)
B(3)-O(1)-K(2)	124.9(8)	La(2)-O(4)-La(3)6	175.7(4)
La(1)-O(2)-La(1)3	109.9(11)	La(2)-O(4)-K(1)6	90.0(3)
La(1)-O(2)-K(3)3	164.2(13)	La(2)-O(4)-K(1)	85.4(3)
La(1)3-O(2)-K(3)3	84.3(6)	La(3)-O(4)-La(2)	166.6(5)
La(3)1-O(2)-La(1)	79.3(7)	La(3)-O(4)-La(3)6	13.19(7)
La(3)1-O(2)-La(1)3	163.1(9)	La(3)6-O(4)-K(1)	91.3(3)

La(3)-O(4)-K(1)6	95.8(3)	La(1)4-O(6)-K(2)4	79.6(2)
La(3)6-O(4)-K(1)6	86.6(3)	La(2)4-O(6)-La(1)4	89.5(3)
La(3)-O(4)-K(1)	84.1(3)	La(2)4-O(6)-K(2)4	87.6(3)
K(1)6-O(4)-K(1)	77.9(2)	La(3)6-O(6)-La(1)4	91.6(3)
B(3)-O(4)-La(2)	91.5(6)	La(3)-O(6)-La(1)4	83.4(3)
B(3)-O(4)-La(3)	91.6(7)	La(3)-O(6)-La(2)4	171.6(5)
B(3)-O(4)-La(3)6	92.7(7)	La(3)6-O(6)-La(2)4	174.1(5)
B(3)-O(4)-K(1)	141.7(9)	La(3)-O(6)-La(3)6	13.09(7)
B(3)-O(4)-K(1)6	140.4(9)	La(3)6-O(6)-K(2)4	86.9(3)
La(3)1-O(5)-K(1)12	159.9(8)	La(3)-O(6)-K(2)4	95.4(3)
La(3)1-O(5)-K(3)3	95.2(8)	B(3)-O(6)-La(1)4	139.6(9)
La(3)1-O(5)-K(3)	108.7(8)	B(3)-O(6)-La(2)4	90.2(7)
K(1)1-O(5)-La(3)1	92.1(7)	B(3)-O(6)-La(3)	92.3(7)
K(1)1-O(5)-K(1)12	70.0(7)	B(3)-O(6)-La(3)6	92.7(7)
K(1)1-O(5)-K(3)3	86.5(7)	B(3)-O(6)-K(2)4	140.8(9)
K(1)1-O(5)-K(3)	107.9(9)	La(1)13-O(7)-La(1)1	177.0(4)
K(3)3-O(5)-K(1)12	92.6(8)	La(1)1-O(7)-K(2)1	78.10(8)
K(3)-O(5)-K(1)12	70.3(6)	La(1)1-O(7)-K(2)13	101.76(8)
K(3)3-O(5)-K(3)	151.1(9)	La(1)13-O(7)-K(2)13	78.10(8)
O(5)3-O(5)-La(3)1	155.3(5)	La(1)13-O(7)-K(2)1	101.76(8)
O(5)3-O(5)-K(1)1	112.3(7)	La(3)14-O(7)-La(1)1	96.5(2)
O(5)3-O(5)-K(1)12	44.3(4)	La(3)15-O(7)-La(1)13	96.5(2)
O(5)3-O(5)-K(3)	68.8(18)	La(3)14-O(7)-La(1)13	86.5(2)
O(5)3-O(5)-K(3)3	82.7(19)	La(3)15-O(7)-La(1)1	86.5(2)
O(9)3-O(5)-La(3)1	42.2(13)	La(3)15-O(7)-La(3)14	14.16(9)
O(9)3-O(5)-K(1)12	157.8(16)	La(3)15-O(7)-K(2)1	96.5(2)
O(9)3-O(5)-K(1)1	127.7(17)	La(3)14-O(7)-K(2)13	96.5(2)
O(9)3-O(5)-K(3)3	77.0(14)	La(3)14-O(7)-K(2)1	88.9(2)
O(9)3-O(5)-K(3)	110.1(16)	La(3)15-O(7)-K(2)13	88.9(2)
O(9)3-O(5)-O(5)3	114.2(15)	K(2)13-O(7)-K(2)1	174.6(4)
B(2)-O(5)-La(3)1	95.7(15)	B(4)-O(7)-La(1)13	88.5(2)
B(2)-O(5)-K(1)1	168.5(17)	B(4)-O(7)-La(1)1	88.5(2)
B(2)-O(5)-K(1)12	103.4(13)	B(4)-O(7)-La(3)14	172.92(5)
B(2)-O(5)-K(3)	77.6(10)	B(4)-O(7)-La(3)15	172.92(5)
B(2)-O(5)-K(3)3	84.4(10)	B(4)-O(7)-K(2)13	87.3(2)
B(2)-O(5)-O(5)3	59.6(13)	B(4)-O(7)-K(2)1	87.3(2)
B(2)-O(5)-O(9)3	56.6(15)	La(1)1-O(8)-K(1)	104.7(4)

La(1)1-O(8)-K(1)6	172.3(4)	La(2)2-O(10)-K(2)16	87.6(4)
La(1)1-O(8)-K(2)1	78.0(2)	La(2)2-O(10)-K(3)4	110.7(6)
La(2)-O(8)-La(1)1	91.0(3)	La(2)2-O(10)-K(3)	116.1(4)
La(2)-O(8)-K(1)6	93.4(3)	K(1)10-O(10)-K(2)2	132.6(6)
La(2)-O(8)-K(1)	92.9(3)	K(1)10-O(10)-K(2)16	88.8(4)
La(2)-O(8)-K(2)1	92.4(3)	K(1)10-O(10)-K(3)	145.5(5)
K(1)-O(8)-K(1)6	81.4(2)	K(1)10-O(10)-K(3)4	69.4(3)
K(1)6-O(8)-K(2)1	95.6(3)	K(2)2-O(10)-K(2)16	137.6(4)
K(1)-O(8)-K(2)1	174.0(3)	K(2)2-O(10)-K(3)4	69.1(3)
B(4)-O(8)-La(1)1	89.1(7)	K(2)2-O(10)-K(3)	75.7(2)
B(4)-O(8)-La(2)	179.0(7)	K(2)16-O(10)-K(3)	70.6(4)
B(4)-O(8)-K(1)6	86.4(6)	K(2)16-O(10)-K(3)4	150.6(4)
B(4)-O(8)-K(1)	88.0(6)	K(3)-O(10)-K(3)4	117.0(4)
B(4)-O(8)-K(2)1	86.6(6)	B(1)-O(10)-La(2)2	174.2(14)
La(3)12-O(9)-La(3)16	2.2(2)	B(1)-O(10)-K(1)10	87.7(7)
La(3)16-O(9)-K(2)2	86.4(7)	B(1)-O(10)-K(2)16	97.3(12)
La(3)12-O(9)-K(2)2	88.4(8)	B(1)-O(10)-K(2)2	93.6(10)
La(3)16-O(9)-K(3)	115.1(9)	B(1)-O(10)-K(3)	68.7(8)
La(3)12-O(9)-K(3)	116.5(11)	B(1)-O(10)-K(3)4	63.5(11)
K(3)-O(9)-K(2)2	78.2(6)	La(1)-O(11)-K(2)2	73.5(3)
O(2)3-O(9)-La(3)12	123(2)	La(1)-O(11)-K(3)4	99.5(3)
O(2)3-O(9)-La(3)16	122(2)	La(1)-O(11)-K(3)	95.9(3)
O(2)3-O(9)-K(2)2	48.0(16)	La(2)-O(11)-La(1)	94.5(3)
O(2)3-O(9)-K(3)	91.5(19)	La(2)-O(11)-K(2)2	165.2(4)
O(2)3-O(9)-O(5)3	128(2)	La(2)-O(11)-K(3)4	99.9(4)
O(2)3-O(9)-B(2)	68(2)	La(2)-O(11)-K(3)	107.3(4)
O(5)3-O(9)-La(3)16	109.3(19)	K(3)4-O(11)-K(2)2	74.2(3)
O(5)3-O(9)-La(3)12	108(2)	K(3)-O(11)-K(2)2	83.1(3)
O(5)3-O(9)-K(2)2	151.6(17)	K(3)-O(11)-K(3)4	147.5(4)
O(5)3-O(9)-K(3)	73.8(14)	B(1)-O(11)-La(1)	169.7(9)
B(2)-O(9)-La(3)12	154.7(19)	B(1)-O(11)-La(2)	95.2(8)
B(2)-O(9)-La(3)16	156.8(17)	B(1)-O(11)-K(2)2	96.4(8)
B(2)-O(9)-K(2)2	111.6(19)	B(1)-O(11)-K(3)	84.5(10)
B(2)-O(9)-K(3)	83.9(12)	B(1)-O(11)-K(3)4	75.4(10)
B(2)-O(9)-O(5)3	61.5(18)	La(2)-B(1)-K(1)10	114.8(4)
La(2)2-O(10)-K(1)10	89.4(3)	La(2)-B(1)-K(2)2	117.5(5)
La(2)2-O(10)-K(2)2	84.7(4)	La(2)-B(1)-K(2)16	117.3(6)

K(1)10-B(1)-K(2)2	112.2(5)	La(3)12-B(2)-La(3)1	176.7(10)
K(1)10-B(1)-K(2)16	77.5(4)	K(3)-B(2)-La(3)12	82.2(2)
K(2)2-B(1)-K(2)16	111.0(4)	K(3)-B(2)-La(3)1	96.6(2)
K(3)-B(1)-La(2)	91.6(4)	K(3)3-B(2)-La(3)1	82.2(2)
K(3)4-B(1)-La(2)	91.0(6)	K(3)3-B(2)-La(3)12	96.6(2)
K(3)-B(1)-K(1)10	142.9(7)	K(3)-B(2)-K(3)3	136.6(10)
K(3)4-B(1)-K(1)10	70.8(4)	O(2)-B(2)-La(3)12	124.9(17)
K(3)4-B(1)-K(2)16	144.4(4)	O(2)3-B(2)-La(3)1	124.9(17)
K(3)-B(1)-K(2)16	67.0(4)	O(2)-B(2)-La(3)1	58.0(14)
K(3)4-B(1)-K(2)2	68.4(4)	O(2)3-B(2)-La(3)12	58.0(14)
K(3)-B(1)-K(2)2	73.0(3)	O(2)3-B(2)-K(3)	80.0(10)
K(3)-B(1)-K(3)4	137.6(6)	O(2)-B(2)-K(3)3	80.0(10)
O(3)-B(1)-La(2)	59.5(8)	O(2)3-B(2)-K(3)3	135.4(11)
O(3)-B(1)-K(1)10	60.6(8)	O(2)-B(2)-K(3)	135.4(11)
O(3)-B(1)-K(2)16	83.0(15)	O(2)3-B(2)-O(2)	86(3)
O(3)-B(1)-K(2)2	163.3(18)	O(2)-B(2)-O(5)3	155.0(15)
O(3)-B(1)-K(3)	122.4(16)	O(2)3-B(2)-O(5)	155.0(15)
O(3)-B(1)-K(3)4	94.9(15)	O(2)-B(2)-O(5)	110.6(18)
O(10)-B(1)-La(2)	176.9(14)	O(2)3-B(2)-O(5)3	110.6(18)
O(10)-B(1)-K(1)10	67.0(6)	O(5)-B(2)-La(3)12	118.8(15)
O(10)-B(1)-K(2)2	63.1(9)	O(5)-B(2)-La(3)1	58.0(11)
O(10)-B(1)-K(2)16	60.2(11)	O(5)3-B(2)-La(3)1	118.8(15)
O(10)-B(1)-K(3)4	92.0(12)	O(5)3-B(2)-La(3)12	58.0(11)
O(10)-B(1)-K(3)	85.7(9)	O(5)3-B(2)-K(3)3	75.1(12)
O(10)-B(1)-O(3)	120.9(14)	O(5)-B(2)-K(3)	75.1(12)
O(10)-B(1)-O(11)	122.2(13)	O(5)-B(2)-K(3)3	67.7(11)
O(11)-B(1)-La(2)	57.8(6)	O(5)3-B(2)-K(3)	67.7(11)
O(11)-B(1)-K(1)10	147.9(14)	O(5)3-B(2)-O(5)	61(3)
O(11)-B(1)-K(2)16	134.5(11)	O(9)3-B(2)-La(3)12	165.4(11)
O(11)-B(1)-K(2)2	60.4(7)	O(9)-B(2)-La(3)1	165.4(11)
O(11)-B(1)-K(3)4	78.0(11)	O(9)3-B(2)-La(3)1	14.9(11)
O(11)-B(1)-K(3)	67.9(8)	O(9)-B(2)-La(3)12	14.9(11)
O(11)-B(1)-O(3)	116.6(12)	O(9)3-B(2)-K(3)	111.2(13)
O(3A)-B(1)-La(2)	67(2)	O(9)-B(2)-K(3)	69.7(11)
O(3A)-B(1)-K(3)	91(4)	O(9)-B(2)-O(2)3	49.3(16)
O(3A)-B(1)-O(10)	112(3)	O(9)3-B(2)-O(2)	49.3(16)
O(3A)-B(1)-O(11)	119(3)	O(9)-B(2)-O(2)	128(3)

O(9)3-B(2)-O(2)3	128(3)	O(6)-B(3)-La(3)6	60.0(6)
O(9)-B(2)-O(5)	120(3)	O(6)-B(3)-La(3)	59.8(6)
O(9)3-B(2)-O(5)	62.0(18)	O(6)-B(3)-K(3)8	87.9(8)
O(9)-B(2)-O(5)3	62.0(18)	O(6)-B(3)-K(3)4	92.2(8)
O(9)3-B(2)-O(5)3	120(3)	O(6)-B(3)-O(4)	118.9(11)
O(9)3-B(2)-O(9)	178(4)	La(1)1-B(4)-La(1)13	129.6(6)
La(2)4-B(3)-La(2)	119.1(4)	La(1)1-B(4)-K(2)1	70.31(19)
La(2)4-B(3)-La(3)6	121.8(4)	La(1)13-B(4)-K(2)13	70.31(19)
La(2)-B(3)-La(3)6	119.1(4)	La(1)13-B(4)-K(2)1	90.3(3)
La(2)-B(3)-K(3)8	96.2(4)	La(1)1-B(4)-K(2)13	90.3(3)
La(2)-B(3)-K(3)4	83.6(3)	K(1)6-B(4)-La(1)1	131.0(3)
La(2)4-B(3)-K(3)8	96.9(4)	K(1)-B(4)-La(1)1	91.56(14)
La(2)4-B(3)-K(3)4	84.2(4)	K(1)-B(4)-La(1)13	131.0(3)
La(3)-B(3)-La(2)4	121.3(4)	K(1)6-B(4)-La(1)13	91.56(14)
La(3)-B(3)-La(2)	118.4(4)	K(1)6-B(4)-K(1)	73.4(4)
La(3)-B(3)-La(3)6	11.32(7)	K(1)6-B(4)-K(2)1	86.54(13)
La(3)6-B(3)-K(3)8	76.8(3)	K(1)-B(4)-K(2)13	86.54(13)
La(3)-B(3)-K(3)4	90.8(4)	K(1)-B(4)-K(2)1	133.2(3)
La(3)-B(3)-K(3)8	88.1(4)	K(1)6-B(4)-K(2)13	133.2(3)
La(3)6-B(3)-K(3)4	102.2(4)	K(2)1-B(4)-K(2)13	134.2(6)
K(3)8-B(3)-K(3)4	178.7(5)	O(7)-B(4)-La(1)13	64.8(3)
O(1)-B(3)-La(2)	59.0(6)	O(7)-B(4)-La(1)1	64.8(3)
O(1)-B(3)-La(2)4	60.3(6)	O(7)-B(4)-K(1)	143.3(2)
O(1)-B(3)-La(3)6	174.7(10)	O(7)-B(4)-K(1)6	143.3(2)
O(1)-B(3)-La(3)	173.2(10)	O(7)-B(4)-K(2)1	67.1(3)
O(1)-B(3)-K(3)8	98.3(8)	O(7)-B(4)-K(2)13	67.1(3)
O(1)-B(3)-K(3)4	82.7(7)	O(8)6-B(4)-La(1)13	64.3(5)
O(1)-B(3)-O(4)	118.7(11)	O(8)-B(4)-La(1)13	149.6(5)
O(1)-B(3)-O(6)	122.3(12)	O(8)-B(4)-La(1)1	64.3(5)
O(4)-B(3)-La(2)	60.0(6)	O(8)6-B(4)-La(1)1	149.6(5)
O(4)-B(3)-La(2)4	179.1(9)	O(8)-B(4)-K(1)	65.4(6)
O(4)-B(3)-La(3)6	59.1(6)	O(8)6-B(4)-K(1)	67.1(7)
O(4)-B(3)-La(3)	59.6(6)	O(8)6-B(4)-K(1)6	65.4(6)
O(4)-B(3)-K(3)8	83.2(8)	O(8)-B(4)-K(1)6	67.1(7)
O(4)-B(3)-K(3)4	95.6(8)	O(8)-B(4)-K(2)13	140.1(5)
O(6)-B(3)-La(2)4	62.0(6)	O(8)-B(4)-K(2)1	67.9(4)
O(6)-B(3)-La(2)	175.5(10)	O(8)6-B(4)-K(2)1	140.1(5)

O(8)-B(4)-K(2)1	67.9(4)	La(2)-O(3A)-K(3)	90.1(16)
O(8)6-B(4)-K(2)1	140.1(5)	K(1)10-O(3A)-K(3)	130.1(18)
O(8)6-B(4)-K(2)13	67.9(4)	K(2)16-O(3A)-K(1)10	86.0(13)
O(8)-B(4)-O(7)	120.2(7)	K(2)16-O(3A)-K(3)	70.3(18)
O(8)6-B(4)-O(7)	120.2(7)	B(1)-O(3A)-La(1)1	157(5)
O(8)6-B(4)-O(8)	119.6(14)	B(1)-O(3A)-La(2)	88(3)
La(1)1-O(3A)-La(2)	86.8(16)	B(1)-O(3A)-K(1)10	79(3)
La(1)1-O(3A)-K(1)10	122.0(18)	B(1)-O(3A)-K(2)16	103(4)
La(1)1-O(3A)-K(2)16	73.2(18)	B(1)-O(3A)-K(3)	65(3)
La(1)1-O(3A)-K(3)	93(3)	O(8)-B(4)-K(2)1	67.9(4)
La(2)-O(3A)-K(1)10	123(3)	O(8)6-B(4)-K(2)1	140.1(5)
La(2)-O(3A)-K(2)16	151(3)		

Symmetry transformations used to generate equivalent atoms:

#1 x,-y-1/2,z+1/2 #2 x+1,y,z #3 -x-1,-y,z

#4 x,-y-1/2,z-1/2 #5 x-1,y,z #6 -x-2,-y-1,z

#7 x,y,z-1 #8 x-1,-y-1/2,z-1/2 #9 -x-1,y-1/2,z-1/2

#10 -x-1,-y-1,z #11 -x-2,-y,z #12 -x-1,y+1/2,z+1/2

#13 -x-2,y-1/2,z+1/2 #14 -x-2,-y-1,z+1 #15 x,y,z+1

#16 x+1,-y-1/2,z+1/2

Table S4. Atomic coordinates equivalent isotropic displacement parameters (Å^2) for K_2. U_{eq} is defined as one-third of the trace of the orthogonalized U_{ij} tensor.

Atoms	*x*	*y*	*z*	U_{eq}
La(1)	10000	7135(1)	9536(2)	17(1)
K(1)	5000	5000	516(17)	52(2)
K(2)	5000	5000	5634(10)	32(1)
Na(1)	0	5000	3060(18)	24(2)
O(1)	10000	3963(9)	6340(20)	19(2)
O(2)	2671(16)	3441(8)	2428(15)	20(2)
O(3)	5000	1844(16)	3660(20)	23(2)
O(4)	10000	5000	9210(30)	19(5)
B(1)	10000	5000	7320(50)	20(5)
B(2)	5000	2898(13)	2770(40)	18(5)

Table S5. Atomic coordinates equivalent isotropic displacement parameters (Å^2) for K_3. U_{eq} is defined as one-third of the trace of the orthogonalized U_{ij} tensor.

Atoms	*x*	*y*	*z*	U_{eq}
La(1)	7066(3)	1138(1)	5397(2)	45(1)
La(2)	8988(1)	3058(1)	3730(1)	17(1)
La(3)	9569(2)	5005(2)	7835(1)	17(1)
K(1)	7399(4)	5216(1)	4305(3)	21(1)
K(2)	12209(4)	1506(2)	5464(4)	34(1)
K(3)	4465(8)	1650(2)	1782(3)	58(1)
O(1)	9194(12)	2565(6)	6263(10)	22(2)
O(2)	6100(40)	338(13)	3980(30)	57(8)
O(3)	5620(20)	3770(11)	3400(30)	46(5)
O(4)	9689(17)	3983(5)	5847(9)	32(2)
O(5)	6030(30)	2(14)	1670(30)	54(7)
O(6)	9542(16)	3479(5)	8212(9)	31(2)
O(7)	10000	5000	321(11)	24(3)
O(8)	9618(15)	4306(5)	2470(8)	27(2)
O(9)	3130(30)	204(15)	2960(30)	48(6)
O(10)	2563(16)	3042(6)	3628(18)	51(3)
O(11)	5635(15)	2415(6)	4215(11)	38(2)
B(1)	4569(19)	3059(8)	3710(20)	26(2)
B(2)	5000	0	2930(30)	29(4)
B(3)	9470(20)	3327(8)	6793(14)	20(3)
B(4)	10000	5000	1753(18)	17(3)
O(3A)	5340(80)	3520(40)	2730(80)	33(12)

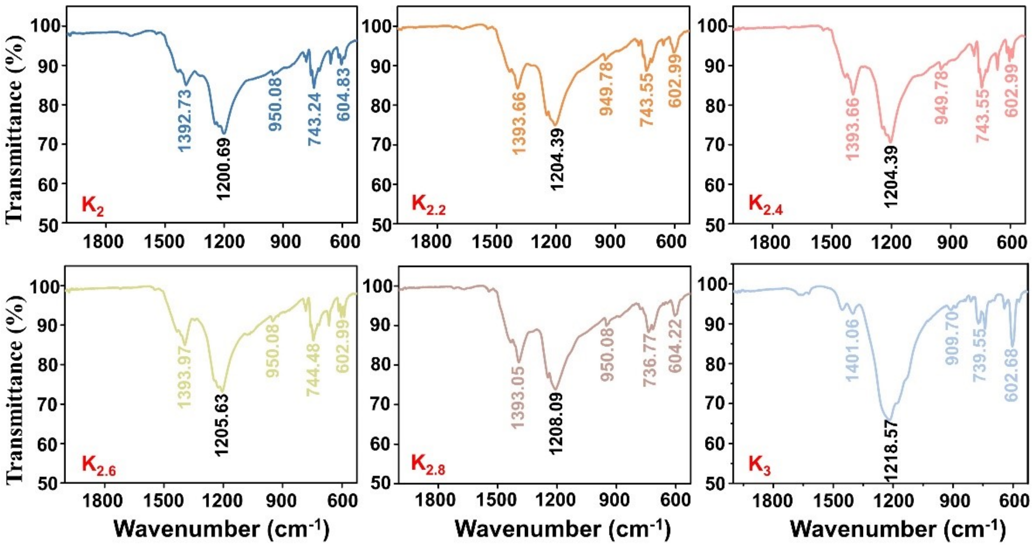

Fig. S1 IR spectrum of the title polycrystalline samples.

Table S6. Comparison of the reported SHG intensity of alkali/alkaline earth metal rare earth borates containing [BO_3] units.

Compounds	Space group	SHG (×KDP)	UV cutoff edge (nm)
$Na_3Gd_2(BO_3)_3$	*Amm*2	1.3	207
$Na_3La_2(BO_3)_3$	*Amm*2	2.0	213
$Na_3La_9O_3(BO_3)_8$	*P*62*m*	6.0	270
$BaYOBO_3$	*P*62*m*	2.6	190
$KNa_2La_2(BO_3)_3$	*Amm*2	2.6	212
$K_5Mg_2La_3(BO_3)_6$	P31m	3.1	200
$Na_3Y_3(BO_3)_4$	*P63mc*	2.4	253
$Na_3Nd_9O_3(BO_3)_8$	*P*62*m*	3.0	230
$YCa_4O(BO_3)$	*Cm*	2.0	220
$LaCa_4O(BO_3)$	*Cm*	3.0	210
$GdCa_4O(BO_3)$	*Cm*	3.4	200
$TmCa_4O(BO_3)$	*Cm*	2.8	254

REFERENCES

1 G. M. Sheldrick, SHELXS-97, Program for the Solution of Crystal Structures, 1997.

2 A. L. Spek, Single-crystal structure validation with the program PLATON, *J. Appl. Crystallogr.*, 2003, **36**, 7-13.

3 S. K. Kurtz and T. T. Perry, A Powder Technique for the Evaluation of Nonlinear Optical Materials, *J. Appl. Phys.*, 1968, **39**, 3798-3813.

4 P. Kubelka and F. Munk, An Article on Optics of Paint Layers, *Z. Tech. Physical*, 1931, **12**, 593.

5 W. Q. Huang, X. Zhang, Y. Q. Li, Y. Zhou, X. Chen, X. Q. Li, F. F. Wu, M. C. Hong, J. H. Luo and S. G. Zhao, A Hybrid Halide Perovskite Birefringent Crystal, *Angew Chem Int Ed*, 2022, **61,** e202202746.

6 S. J. Clark, M. D. Segall, C. J. Pickard, P. J. Hasnip, M. J. Probert, K. Refson and M. C. Payne, First principles methods using CASTEP, *Z. Kristallogr.*, 2005, **220**, 567-570.

7 M. Ernzerhof and G. E. Scuseria, Assessment of the Perdew-Burke-Ernzerhof exchange-correlation functional, *J. Chem. Phys.*, 1999, **110**, 5029-5036.
